# Heavy Metal Tolerance of Microorganisms Isolated from Coastal Marine Sediments and Their Lead Removal Potential

**DOI:** 10.3390/microorganisms11112708

**Published:** 2023-11-04

**Authors:** Katleen L. Alvarado-Campo, Marynes Quintero, Bernarda Cuadrado-Cano, Manuela Montoya-Giraldo, Elver Luis Otero-Tejada, Lina Blandón, Olga Sánchez, Ana Zuleta-Correa, Javier Gómez-León

**Affiliations:** 1 Marine Bioprospecting Line, Evaluation and Use of Marine and Coastal Resources Program–VAR, Marine and Coastal Research Institute–INVEMAR, Santa Marta 470006, Magdalena, Colombia; katleen.alvarado@invemar.org.co (K.L.A.-C.); marynes.quintero@invemar.org.co (M.Q.); elverotero@sophicol.org (E.L.O.-T.); lmblando@unal.edu.co (L.B.); javier.gomez@invemar.org.co (J.G.-L.); 2 Master’s Program in Microbiology, College of Medicine, Universidad de Cartagena, Cartagena de Indias 130014, Bolívar, Colombia; bcuadradoc@unicartagena.edu.co; 3 Department of Genetics and Microbiology, Faculty of Biosciences, Universitat Autònoma de Barcelona, 08193 Bellaterra, Spain; olga.sanchez@uab.cat

**Keywords:** marine bacteria, marine fungi, cadmium, zinc, *Enterobacter* sp., *Stenotrophomonas* sp., *Pseudomonas* sp.

## Abstract

In this study, 338 microorganisms, comprising 271 bacteria and 67 fungi, were isolated from sediment samples collected from underexplored Pacific and Caribbean regions of Colombia. Screening trials were conducted on selected strains (*n* = 276) to assess their tolerance to cadmium (Cd^2+^), lead (Pb^2+^), and zinc (Zn^2+^), leading to the identification of six bacteria capable of withstanding 750 mg·L^−1^ of each heavy metal ion. Three promising microorganisms, identified as *Enterobacter* sp. INV PRT213, *Pseudomonas* sp. INV PRT215, and *Stenotrophomonas* sp. INV PRT216 were selected for lead removal experiments using LB broth medium supplemented with 400 mg·L^−1^ Pb^2+^. Among these, *Pseudomonas* sp. INV PRT215 exhibited significant potential, removing 49% of initial Pb^2+^ after 240 min of exposure (16.7 g wet biomass·L^−1^, pH 5, 30 °C). Infrared spectra of Pb-exposed biomass showed changes in functional groups, including carbonyl groups of amides, carboxylate, phosphate, hydroxyl, and amine groups, compared to the not-exposed control. These changes suggested interactions between the metal and functional groups in the biomass. The findings of this study highlight the potential of microorganisms derived from coastal marine environments as promising candidates for future applications in bioremediation of polluted environments contaminated with heavy metals.

## 1. Introduction

Metal ions reach ecosystems through various natural processes and anthropogenic activities [1]. The leading causes of their natural release from the lithosphere and subsequent accumulation in ecosystems include erosion, volcanic activity, and forest fires, from which these metals undergo accumulation cycles without causing adverse effects on the environment [2]. On the other hand, several human activities extensively use metals such as lead, cadmium, molybdenum, vanadium, and chromium to manufacture multiple products, including batteries [3], plastic [4], projectile lubricants, insecticides, ceramics, paint and glass pigments [5], photography [6], welding, wood conservation agents, and tanning agents, and in the chemical industry as a whole [7]. The frequent use of metals in industrial settings is closely linked to environmental pollution due to inadequate residue management that accelerates metal accumulation in sediments and water-receiving bodies [1].

The development of suitable alternatives to mitigate heavy metal pollution and environmental deterioration is the focus of different research fields [8,9]. The presence of these toxic agents in the environment generally induces morphological and physiological changes in microbial communities. Therefore, polluted environments are considered a source of promising metal-resistant strains helpful in the development of new bioremediation methods, which are commonly regarded as cost-effective, ecological, and efficient technologies [10,11]. There is evidence of organisms and microorganisms that, due to their metabolic capabilities, can tolerate and/or transform metals for their benefit, accumulating them within themselves [12]. In this sense, microorganisms or their metabolic products have been employed to transform toxic compounds into less harmful forms, thus tackling environmental damage [13,14].

Microorganisms employ several mechanisms for bioremediation of soil and water polluted with heavy metals including the secretion of extracellular barriers, extracellular and intracellular sequestration, active transport of metal ions, production of metal chelators, and enzymatic detoxification [15,16]. The permeability barrier involves the production of exopolysaccharides and biofilm formation to physically sequester and chemically modify metal ions into less toxic molecules [17]. On the other hand, the extracellular sequestration process is a passive uptake mechanism that involves the accumulation of metal ions in the cellular periplasm or the complexation of metal ions into insoluble compounds, causing metal precipitation. Intracellular sequestration implicates their transport within the cell, and, therefore, it requires interaction of metal ions with cell-surface ligands [18]. Active transport of metal ions is based on either ATP hydrolysis or an electrochemical gradient comprising the participation of ABC transporter proteins, cation diffusion facilitator (CDF), and P-type ATPases, among other proteins involved in metal tolerance [19]. Several reports indicate the production of microbial metabolites acting as chelating agents, as well as the formation of molecules such as metallothioneins, phytochelatins, and biosurfactants during heavy metal detoxification. The production of enzymes that can biologically transform or chemically modify heavy metal ions in less toxic forms is also frequently reported [15].

The use of autochthonous bacteria and fungi for ecosystem remediation has become one of the most critical activities when designing biotechnological processes to restore affected environments [7]. This is especially significant for organisms from marine-coastal environments, which exhibit adaptability to a wide range of temperature, pressure, and high salinity gradients, allowing them to thrive under diverse conditions without cell damage [14,20].

The Northern Pacific and the southern area of the Atlantic (Gulf of Urabá) are considered essential areas of Colombia, known for their high biodiversity and abundant water resources [21]. The Atrato River, on the Caribbean side, stands as one of the world’s fastest-flowing rivers, while the Pacific region boasts waterways of great importance for the country’s trade, as is the case of the Baudó and San Juan rivers. However, despite their economic and ecosystem significance, these rivers face concerning levels of heavy metal pollution attributed to illegal gold extraction and untreated wastewater dumping, which resulted in the accumulation of these toxic compounds into the sediments, particularly in the Colombian Pacific area [22]. For example, cadmium pollution is often linked with the use of agrochemicals, of battery manufacturing, pigments, anticorrosive agents, and contaminated irrigation water [23,24,25]; zinc contamination may result from discharge of dyes and detergents, while lead is mainly derived from the textile industry, battery production, paint manufacturing, insecticide use, fossil fuel consumption, waste incineration, and metallurgical processes [26,27]. In particular, lead represents one of the most significant heavy metal contaminants on a global scale, raising alarming concerns on both human health and biodiversity [17]. Distribution reports in Colombia indicate the presence of this metal across different environmental matrices, posing a public health risk [5,28]. In this context, there has been a growing interest in developing eco-friendly alternatives to treat lead-polluted environments, particularly microbial-based technologies that promote the sustainability of the treatment process.

Research focused on studying the microbial diversity of environments affected by heavy metal pollution is scarce, and even more so are studies that evaluate microbial diversity associated with river mouth sediments in Colombia. Still, the few existing reports suggest an increased abundance of metal-resistant species due to challenging conditions [29]. Consequently, this study aimed to enhance the knowledge of Colombian microbial diversity from underexplored environments such as coastal marine sediments. It also sought to evaluate the potential of isolated microorganisms to withstand various heavy metals, with a special emphasis on lead removal. The results of this work contribute to global efforts to broaden our knowledge about microbial biodiversity from marine and coastal environments and evaluate its ability to bioremediate widespread pollutants such as heavy metals.

## 2. Materials and Methods

### 2.1. Study Area and Sample Collection

**Figure 1:**
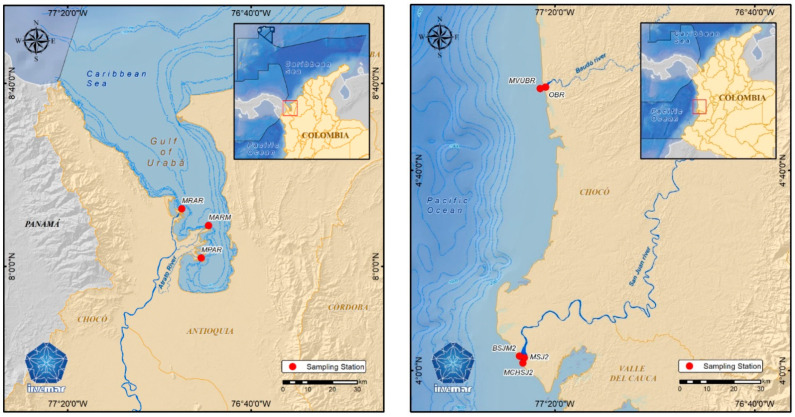
Sampling sites for the collection of estuarine sediments in the Colombian South Atlantic (**left**) and North Pacific Sea (**right**). Map created in the Information Services Laboratory (LABSIS) at Marine and Coastal Research Institute ‘José Benito Vives de Andréis’ (INVEMAR) using the ArcGIS 10.8 software.

Three sampling zones (eight total sampling sites) in the Chocó region of Colombia were selected for collecting sediment samples (Figure 1). These sites experience anthropogenic influence due to the discharge of untreated wastewater and alluvial gold mining [30,31,32]. Three sampling sites were located in estuarine areas in the southern Atlantic portion of the Colombian Caribbean Sea, i.e., in the Atrato River mouth (Gulf of Urabá) (Figure 1, left). Sampling site details are given in Table 1. The remaining sampling zones were located to the north of the Colombian Pacific Sea in the San Juan River mouth and Baudó River mouth (Figure 1, right). 

A total of 200 g of sediments were collected from each sampling site using Van Veen dredges with a surface of 0.08 m^2^. The sediments were stored in Whirl-Pak^®^ sterile sample bags and maintained at 4 °C until they were processed in the Marine Bioprospecting Laboratory of the Marine and Coastal Research Institute ‘José Benito Vives de Andréis’ (INVEMAR). Additionally, physicochemical parameters (temperature, salinity, and pH) were measured in the surface water of each sampling site.

### 2.2. Microorganism Isolation and Culture

Microorganisms were isolated in Petri dishes using different culture media including starch casein agar (SCA), International *Streptomyces* project 2 (ISP2) medium, ISP4 medium, ISP3 medium, marine agar (MA), tryptic soy agar (TSA), Actinobacteria agar (AA) Boyd and Kohlmeyer (B&K) medium, malt extract agar, and Rose Bengal Chloramphenicol agar (RBC). Components of all culture media are shown in (Table S1). Three isolation methodologies were followed for sediment processing: (1) a selective pressure method using mercury chloride (HgCl_2_) to isolate mercury-resistant strains, (2) a phenol pretreatment to isolate sporulated microbes, and (3) a direct culture of non-pretreated sediments. For the first method, 5.0 g of each sediment sample was mixed with 10 mL of Luria Bertani-LB broth [33] and supplemented with 5.0 mg·L^−1^ of mercury chloride. The test tubes were incubated in a Scientific Industries SI™ Roto-Shaker for up to 60 min at room temperature (22 °C) [34]. For the phenol pretreatment, a total of 20 g of each sediment sample was added to 100 mL of 1.5% (*w*/*v*) phenol [35]. The mixture was incubated in an orbital shaker at 140 rpm and 30 °C for 60 min. Lastly, a sterile cotton swap was used to directly inoculate non-pretreated and pretreated sediments on the surface of different culture media (Table S1). After inoculation, plates were incubated at 25–30 °C in the dark for three to five days. In order to obtain individual colonies, serial dilutions 1/10 and 1/10^2^ were carried out in test tubes containing 9.0 mL of dilution water, and aliquots of 0.1 mL were inoculated via spread plating in the culture media. Obtained isolates were phenotypically characterized and the cultivable strains were quantified by means of the colony forming unit by gram (CFU·g^−1^). The obtained axenic cultures were cryopreserved at −80 °C. All microorganisms were deposited in the culture collection of the Marine Natural History Museum of Colombia –Makuriwa located in INVEMAR, with consecutive numbers ranging from 76844 to 77182. Once microorganisms were identified, they were assigned a catalog number (i.e., INV PRT 211, INV PRT212, INV PRT213, INV PRT215, and INV PRT216).

### 2.3. Cadmium (Cd^2+^), Lead (Pb^2+^), and Zinc (Zn^2+^) Tolerance Screening

A qualitative heavy metal tolerance screening was performed by exposing the microorganisms to varying concentrations of Cadmium (Cd^2+^), Lead (Pb^2+^), and Zinc (Zn^2+^). Tolerance screening was performed using 276 selected microorganisms out of 338 isolated colonies. The selection was based on two criteria (a) microorganisms exhibited a rapid growth after 24 to 48 h of incubation, and (b) colonies had a creamy appearance that allowed for easy manipulation and culture. Screened microorganisms (*n* = 276) included 21 yeast and 255 bacteria.

This tolerance screening was performed via the agar diffusion technique following the modified methodology previously reported [36]. Briefly, massive culturing of the microorganisms was carried out on Luria Bertani agar (LB) marked with non-perforated equidistant wells (diameter = 9 mm). Liquid standard solutions (Certipur^®^ Merck, Darmstadt, Germany) of the heavy metals nitrate salts (i.e., Cd(NO_3_)_2_, Pb(NO_3_)_2_, and Zn(NO_3_)_2_, adjusted at a pH 6.0, 5.0 and 6.0, respectively) were placed on the marked wells at concentrations of 50, 150, 250, and 350 mg·L^−1^. Inoculated plates were incubated at 30 °C for 48 h. All experiments were carried out in triplicate. A microorganism was catalogued as tolerant (+) if growth was observed after 24–48 h of culture; not tolerant (−) if growth was inhibited entirely around wells, or sensitive (±) if some colonies were formed [36]. 

Microorganisms exhibiting growth at the maximum concentration level (i.e., 350 mg·L^−1^) of all tested heavy metals were subjected to further tolerance screening trials at increased concentrations of Pb^2+^, Cd^2+^ and Zn^2+^ ions. Lead and zinc were evaluated at 450, 550, 650, and 750 mg·L^−1^, while Cd^2+^ ion was evaluated at 550 and 750 mg·L^−1^.

### 2.4. Molecular Identification of Promising Bacteria

Microorganisms (*n* = 6) that showed appropriate growth at a concentration of 750 mg·L^−1^ of cadmium, lead, and zinc during the qualitative tolerance screening were selected for taxonomic classification. Briefly, the bacterial DNA was extracted using the Ultraclean Tissue & Cells (MoBio Laboratories Inc., Carlsbad, CA, USA) and Wizard^®^ Genomic DNA purification (Promega, Madison, WI, USA) kits while following the manufacturers’ instructions. DNA purity was determined via a NanoDrop 2000 UV-Vis spectrophotometer (ThermoFisher Scientific™, Waltham, MA, USA). The amplification of the bacterial 16S rRNA gene was carried out via PCR reactions in a BioRad T100™ thermal cycler (Hercules, CA, USA) with a final volume of 25 μL, which contained the universal primers (0.5 µL) 27F (5’ AGA GTT TGA TCM TGG CTC AG 3’)-(0.5 µL) 1492R (5′-GGT TAC CTT GTT ACG ACT T-3′), a 1X polymerase buffer (5.0 µL), 12.0 µL of sterile MilliQ water, 0.5 µL of Taq polymerase (Bioline, Memphis, TN, USA), and 6.0 µL of DNA [37,38]. The PCR products amplification was verified by means of horizontal electrophoresis in 1% agar gel, using the GelRed^®^ (Biotium, Inc., Hayward, CA, USA) revealing reagent and a molecular weight marker of 50 pb (HyperLadder™—Bioline, London, UK). The gels were documented via UV light exposure in the ENDURO™ GDS (Edison, NJ, USA)—LABNET digital capture system. The amplified products were sent to Macrogen (Seoul, Korea) for sequencing using the Sanger/capillary method. The final sequences were analyzed and compared with those available in the Refseq_rna (Reference RNA Sequences) database of the National Center for Biotechnology Information (NCBI, https://www.ncbi.nlm.nih.gov/ accessed on 8 September 2023). Alignment was performed using MUSCLE (Multiple Sequence Comparison by Log-Expectation). Phylogenetic analysis was conducted in the MEGA-X software, version 10.0.5, using the maximum likelihood test with 1000 Bootstrap replicates and based on the Kimura 2 evolution model, with a Gamma distribution (G) and six discrete Gamma categories. The 16S rRNA gene sequences were deposited in GenBank database with the accession numbers OR557202, OR557203, OR557204, OR557205 and OR557206. The bacteria with consecutive number 77050 could not be identified due to low-quality of the 16S rRNA gene sequence.

### 2.5. Viability Test of Promising Bacteria

Three bacteria, identified as *Enterobacter* sp. INV PRT213, *Pseudomonas* sp. INV PRT215, and *Stenotrophomonas* sp. INV PRT216 were selected to verify their viability through a resazurin test. This test was performed in 96-well plates and evaluated concentrations up to 500 mg·L^−1^ of the three metal ions. To this effect, the bacteria were cultivated in a LB medium supplemented with Cd^2+^, Pb^2+^ and Zn^2+^ salts at increasing concentrations of 10, 50, 100, 150, 200, 250, 350, 450, and 500 mg·L^−1^. Growth controls were prepared, including (1) the microorganism without metal, (2) the no inoculated LB medium (abiotic control), and (3) the metal solutions (abiotic control). The plates were incubated at 30 °C for 72 h in a wet chamber to avoid evaporation. A volume of 2.0 µL of resazurin was added to each well (0.4 mg·L^−1^), and spectrophotometric readings were performed at 570 and 603 nm [39,40]. The viability percentage was calculated following Equation (1):

(1) $$Viability(\%)=\frac{\left(A_{LW}-(A_{HW}\times Ro)\;treated\;sample\right)}{\left(A_{LW}-(A_{HW}\times Ro)\;growth\;control\right)}$$

where:

*A_LW_* = Absorbance to the lowest wavelength minus the blank medium

*A_HW_* = Absorbance to the highest wavelength minus the blank medium

$$Correction\;factor\;(Ro)=\frac{A_{LW}}{A_{HW}}$$

### 2.6. Bacterial Growth Curves of Promising Isolates at Different Pb^2+^ Concentrations

Liquid cultures of three bacterial isolates (*Enterobacter* sp. INV PRT213, *Pseudomonas* sp. INV PRT215 and *Stenotrophomonas* sp. INV PRT216) that remained viable at 500 mg·L^−1^ of Cd^2+^, Pb^2+^ y Zn^2+^ were completed in 100 mL flasks (25 mL working volume). Flasks contained LB broth at a pH of 5.0, supplemented with concentrations of 0 (control), 10, 100, 200, 300, and 400 mg·L^−1^ of the metal (Pb(NO_3_)_2_). A volume of 1 mL of the inoculum adjusted to the 0.5 McFarland scale was added to each culture, and the flasks were incubated at 30 °C in an orbital shaker-incubator (Thermo Scientific™, Waltham, MA, USA) at 140 rpm for 144 h [41]. Growth was measured at 0, 24, 48, 72, 96, 120, and 144 h, using 300 µL aliquots to measure the optical density (OD_600nm_) with a Thermo Scientific™ spectrophotometer and the Multiskan Go software version 4.1. The assays were carried out in triplicate.

### 2.7. Pb^2+^ Removal Experiments

Experiments were carried out to evaluate the potential of three bacterial isolates (*Enterobacter* sp. INV PRT213, *Pseudomonas* sp. INV PRT215, and *Stenotrophomonas* sp. INV PRT216) to remove lead from an aqueous solution. Lead (Pb^2+^) removal trials were performed at three initial wet bacterial biomass values (8.3, 16.7, and 25.0 g·L^−1^) and two exposure periods (120 and 240 min). Experiments were completed in 100 mL flasks containing 60 mL of LB broth (pH = 5.0, adjusted with 0.5 M nitric acid) and supplemented with 400 mg Pb^2+^·L^−1^ (as Pb(NO_3_)_2_). Flasks were incubated in an orbital shaker (Thermo Scientific™) at 30 °C and 160 rpm and sampled after 120 and 240 min of exposure. Abiotic controls of the solution (400 mg·L^−1^ Pb^2+^) were included and incubated under the same experimental conditions. All assays were carried out in triplicate. After incubation, flask contents were centrifuged at 4000 rpm and 4 °C for 45 min (Hettich Universal 320 centrifuge) to separate biomass. The supernatants were acidified with a 65% HNO_3_ solution (Merck^®^) until a pH ≤ 2 was reached and stored at 4 °C until quantification of the total Pb^2+^ (mg Pb·L^−1^) in the liquid phase.

The extent of Pb^2+^ metal ions removal was quantitatively evaluated via air-acetylene direct flame atomic absorption spectrophotometry (AAS) [41]. The samples were processed following the SM 3030E and SM 3111B methods from [42]. An AT Thermo S Series iCE-330 spectrophotometer was implemented to analyze the samples using a calibration curve based on the standard addition method. The data were recorded as the mean plus the standard deviation [41].

The total Pb^2+^ removal percentage (*R%*) was calculated using Equation (2) [43]:

(2) $$R(\%)=\frac{C_{i}-C_{f}}{C_{i}}\times 100$$

where:

C_i_: Pb (II) concentration in the abiotic control

C_f_: final concentration of Pb (II) in cell-free supernatants

### 2.8. Fourier Transform Infrared (FT-IR) Spectroscopy Analysis of Bacterial Biomass

A Fourier transform infrared (FT-IR) spectroscopy analysis aimed to determine the possible cell surface changes associated with Pb^2+^ removal capacity [44]. For this purpose, 2 mL aliquots were taken from experimental units showing the highest removal percentage. These samples were centrifuged at 4000 rpm and 4 °C for 40 min. The resulting biomass was washed four times with a saline solution at 0.8% (*w*/*v*) and concentrated in a Speedvac Thermo Scientific™ (Waltham, MA, USA) SPD111V concentrator for 4 h at 80 °C. The dry biomass was macerated in a mortar until a fine powder was obtained and mixed with anhydrous potassium bromide (KBr) at a proportion of 1:100 to obtain a pellet of the mix through a pellet press (PIKE). The biomass cultivated without Pb^2+^ salt was used as a negative control. A Shimadzu infrared spectrophotometer (IR-TRACER-100) (Kyoto, Japan) with a DLATGS detector (with temperature control for the far/medium infrared) was implemented to obtain spectra in the 400–4000 cm^−1^ wavenumber range, with 28 scans and a 4 cm^−1^ resolution.

### 2.9. Statistical Analysis

The data are presented as average values, with bars representing their standard deviation. A descriptive analysis of the data was conducted. These data corresponded to the number of strains isolated in each sampling site and those that tolerated the different heavy metals, and they were presented as percentages (%). A nonlinear regression was performed using four parameters in the RStudio statistical software version 4.3.3 [45] to calculate the maximum concentration at which the strains remained 80% viable in the presence of the metals. The removal percentages were compared through a one- or two-way analysis of variance (ANOVA) following the methodology described by [46] and using the Statistics Kingdom online free software (https://www.statskingdom.com/ accessed on 31 May 2023).

## 3. Results

### 3.1. Study Area and Sample Collection

Signs of pollution were observed in all locations, including the presence of foam and solid waste such as various plastics, food packaging, and textiles, among others. The environmental conditions of the sampling sites were influenced by the rainy season of the Chocó region. The shades of color in the surface waters were light brown, dark brown, and greyish green. However, surface water samples were odorless except for those at MVUBR and MCHSJ2 sites, which reported a slight sulfur and a woody odor, respectively. The physicochemical parameters of the surface water in the sampling sites are shown in Table 1. The salinity oscillated between 0 (below detection limit) and 7.5 ppt with the MPAR (7.5), MVUBR (7.2), and OBR (6.5) sites in the Atrato and Baudó river mouths exhibiting the highest values. In contrast, MRAR (0.0), MARM (0.2), and MCHSJ2 (0.3) collection sites in the Atrato and San Juan River mouths showed the lowest salinity values. Typical values for estuarine salinity range from 0.5 to 17 ppt. The salinity of brackish water exhibits a wide range since it can rapidly change depending on tides, weather, and other factors [47]. Most of the salinity values in this study fell within the typical range, while some of the observed lower values can be attributed to dilution events during the rainy season. The surface water temperature oscillated between 25.6 and 29.7 °C. Collected sediments from all sites were silt and clay with a dark brown color and collected from depths ranging from 0.5 to 7.6 m.

### 3.2. Microorganism Isolation and Culture

A total of 338 strains were isolated from collected sediment samples, comprising 67 fungi and 271 bacteria. Phenotypical characterizations revealed bacterial colonies of various morphologies such as circular, filamentous, and irregular shapes, along with smooth, glistening, and rough surfaces. The elevations ranged from raised to convex, flat, umbonate, and crateriform. The edges of the colonies exhibited entire, undulate, curled, and lobate features, and they had creamy, mucilaginous, or dry consistencies. Additionally, some isolated samples showed pigmented colonies and the production of extracellular pigments, as well as iridescent colonies with pulverulent consistency. As for the isolated fungi, culture examinations showed cottony fluffy and filamentous colonies in addition to yeast forms (Figure S1). Regarding microscopic characteristics, three distinct bacteria shapes were observed: Gram-positive and Gram-negative bacilli, Gram-positive filamentous bacteria, and Gram-positive cocci. On the other hand, hyaline septate and non-septate fungal hyphae and yeast forms were observed for the fungal strains.

**Figure 2:**
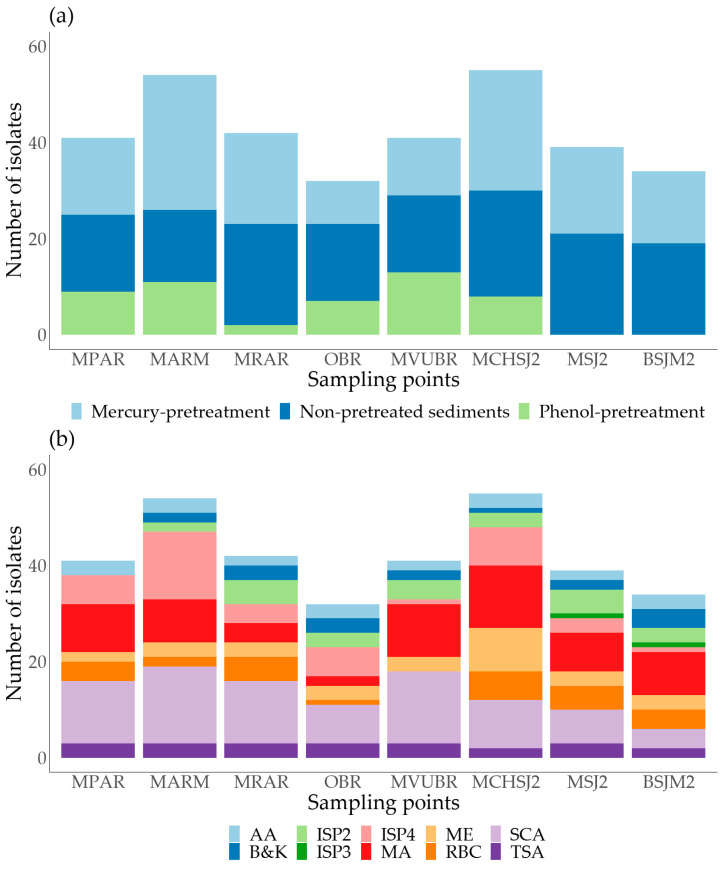
Number of isolates from sediment samples collected from marine environments. (**a**) Sediment processing method and (**b**) Culture medium.

Among the total isolated microorganisms, 137 strains (40.5%) were retrieved from the Atrato River mouth, 128 (37.9%) from the San Juan River mouth, and 73 (21.6%) from the Baudó River mouth (Figure 2a). It is important to note that the lower number of isolates in the Baudó region can be due to the fewer collection sites in this area (i.e., two sampling sites) compared to other collection stations (i.e., three sampling sites). Zone inaccessibility was the main reason for the discrepancy.

The non-pretreated sediments allowed for the highest recovery of cultivable microbes, totaling 146 strains (43.2%). Among these, 62 were isolated from the San Juan River mouth, 52 from the Atrato River mouth, and 32 from the Baudó River mouth. These results were expected as no stressors restricted microbial growth, allowing for a higher proliferation rate of vegetative cells. On the other hand, challenging conditions, such as those imposed by the phenol pretreatment, resulted in lower isolate numbers (50 isolates, representing 14.8% of total strains). Phenol-treated sediments selectively supported sporulated slow-growth organisms like Actinomycetota, which are frequently outpaced by vegetative cells during solid culture. These microorganisms were mainly obtained from the Atrato and Baudó regions, as microbial growth was not detected (<1 CFU·g^−1^) in phenol-pretreated samples from two San Juan River mouth sites. Regarding mercury chloride pretreated samples, 142 cultivable microorganisms (42%) were obtained from the sampling sites: 63, 58, and 21 from the Atrato, San Juan, and Baudó river mouths, respectively. It is possible that the mercury pretreatment represented a milder pressure condition that may more closely resemble the natural environment from which these microorganisms were isolated, given that the sampling areas were characterized by the presence of mercury resulting from illegal mining processes [22,30].

Culture media such as SCA, ISP4, and MA strongly supported the isolation of microorganisms, accounting for 57.7% (*n* = 195) of the total isolates in this work. Notably, the SCA medium accounted for 25.4% (*n* = 86) of the recovered cultivable strains. The SCA culture media formulation contains complex carbon sources such as starch and a high salt content. This composition could promote the development of several microorganisms, which may explain the higher number of isolates obtained using this medium. Marine agar was the most suitable culture medium for colonies with a creamy consistency, pigmentation, or other interesting phenotypic characteristics, constituting 19.5% (*n* = 66) of the total isolates. The remaining 13.6% (*n* = 46) strains were isolated from ISP2, ISP3, Actinobacteria agar (AA), RBC, TSA, malt extract, and Boyd and Kohlmeyer (B&K) medium (Figure 2b).

### 3.3. Cd, Pb, and Zn Tolerance Screening

Heavy metal tolerance screening was performed using 276 selected microorganisms out of 338 isolated colonies. The selection was based on rapid growth and suitability for spectrophotometric measurements. Consequently, some mycelial fungi and Actinomycetota were excluded due to cell aggregate formation that interfered with reproducible data collection.

**Figure 3:**
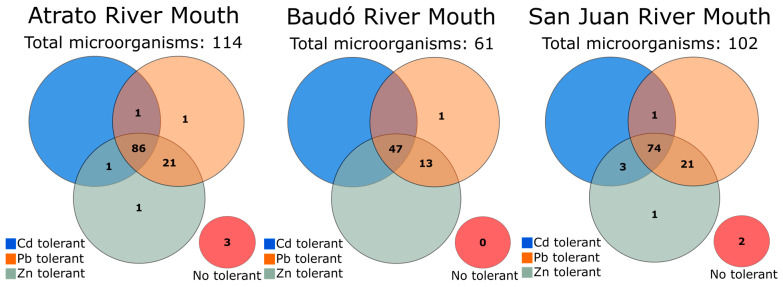
Number of tolerant isolates by sampling site. Tolerance screening was performed at varying concentrations (50, 150, 250, and 350 mg·L^−1^) of cadmium, lead, and zinc. A microorganism was considered tolerant if it exhibited growth at any concentration level of a given metal.

Screened microorganisms included 21 yeast and 255 bacteria (Table S2). Tolerance to the metals was observed in microorganisms with different morphologies, consistencies, and textures (creamy and sporulated). Some bacteria excreted mucilaginous substances in the presence of 150–350 mg·L^−1^ Cd^2+^ (76955) (Figure S2). In addition, the culture of microorganism 76955 excreted a brown pigment when exposed to Cd^2+^ and Zn^2+^. These could be exopolysaccharides (EPS) widely reported as molecules excreted by different organisms under stress conditions to chelate metal ions [48]. Most evaluated organisms showed some level of tolerance to at least one heavy metal. Only five bacteria (catalog numbers 76851, 76852, 76975, 76997, and 77085) exhibited complete inhibition at all concentration levels of the three individually assessed heavy metal ions (Cd^2+^, Pb^2+^, and Zn^2+^) (Figure 3). It was observed that the samples isolated from the Atrato River mouth reported most of the tolerant strains (*n* = 111), followed by those from the San Juan River mouth (*n* = 100) and the Baudó River mouth (*n* = 61) (Figure 3). A total of 121, 251, and 214 microorganisms displayed colony development at some level of cadmium, lead, or zinc, respectively (Figure S3). Among microbial groups, bacteria exhibited higher tolerance to the maximum concentrations of Cd, Pb, and Zn, with 106 bacteria growing at 350 mg·L^−1^ of each heavy metal, while only 4 yeasts (fungal) isolates developed colonies at this concentration for all 3 metals. Isolated yeasts were particularly susceptible to cadmium, with only eight cultures (38% of fungal species) growing at concentrations ≥ 150 mg·L^−1^. However, all of them (except yeast 77149) were tolerant to some level of lead and zinc.

Among these 110 microorganisms, eleven strains were selected based on growth rate and ease of handling (creamy colonies) (Table S3). Additional screening experiments allowed the identification of six microorganisms tolerant to concentrations of up to 750 mg·L^−1^ of Cd^2+^, Pb^2+^ y Zn^2+^ ions. Five of these were isolated via direct swabbing (INV PRT212, INV PRT213, 77050, INV PRT215, and INV PRT216), and one (INV PRT211) resulted from the mercury chloride pretreatment. The six promising bacteria were subjected to molecular identification procedures (see Section 3.4).

### 3.4. Molecular Identification and Phylogenetic Analysis

Six bacteria tolerant to concentrations of up to 750 mg·L^−1^ of Cd^2+^, Pb^2+^ and Zn^2+^ ions were selected for molecular analyses and taxonomic classification. The sequencing data from one of the samples (bacteria 77050) did not meet the minimal quality requirements, making it impossible to assign this microorganism to a taxonomic group.

**Figure 4:**
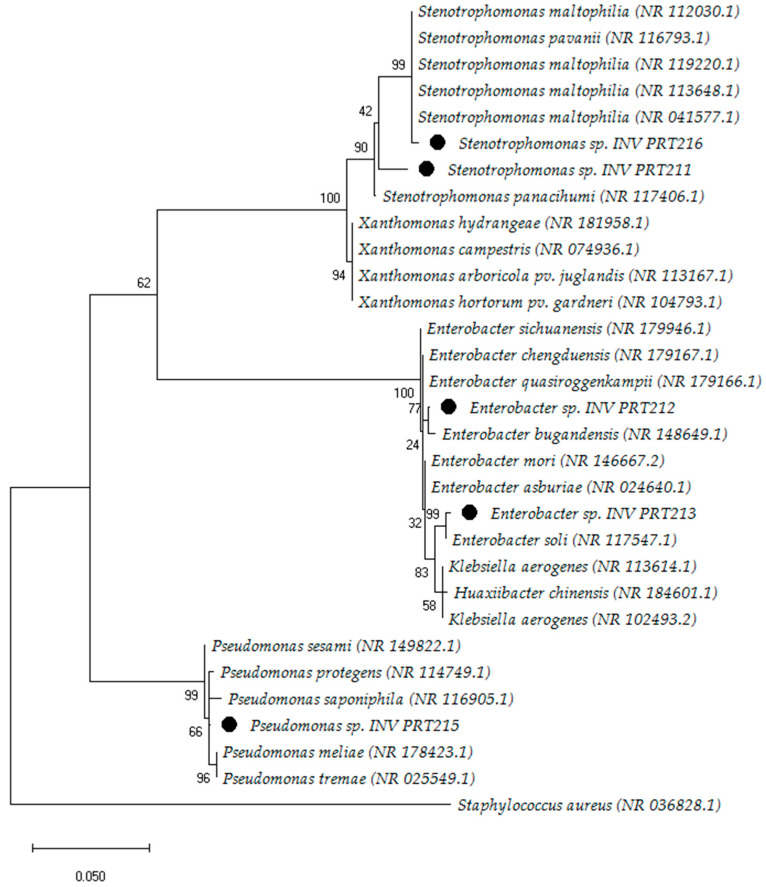
Phylogenetic tree of five heavy-metal-tolerant bacteria isolated from marine sediments. The tree was constructed using the MEGA-X software, version 10.0.5. Based on the maximum likelihood analysis of the 16S rRNA sequences, the evaluated microorganisms were identified as *Stenotrophomonas* sp. INV PRT211, *Enterobacter* sp. INV PRT212, *Enterobacter* sp. INV PRT213, *Pseudomonas* sp. INV PRT215, and *Stenotrophomonas* sp. INV PRT216.

The resulting phylogenetic tree comprised three clades, each representing species closely related to the evaluated microorganisms. These bacteria were classified within the phylum Pseudomonadota, previously denoted as Proteobacteria [49] (Figure 4). Based on the analysis of 16S rRNA sequences, we identified the genera *Stenotrophomonas* (INV PRT211 and INV PRT216), *Enterobacter* (INV PRT212 and INV PRT213), and *Pseudomonas* sp. INV PRT215. Identity percentages for these bacteria ranged between 97 and 99% when compared to the strains reported in the NCBI’s Reference RNA Sequences database (Table S4).

Further experiments were conducted using three out of five molecularly identified microorganisms: INV PRT213, INV PRT215, and INV PRT216. The selection was based on rapid growth after 24 h of culture and isolation region: INV PRT213, INV PRT215, and INV PRT216 were isolated from the Atrato, San Juan, and Baudó regions, respectively.

### 3.5. Viability Test of Enterobacter sp. INV PRT213, Pseudomonas sp. INV PRT215, and Stenotrophomonas sp. INV PRT216

**Figure 5:**
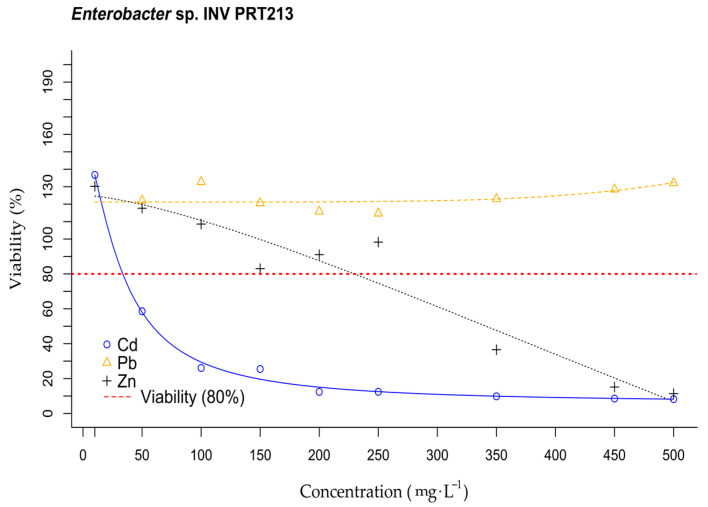

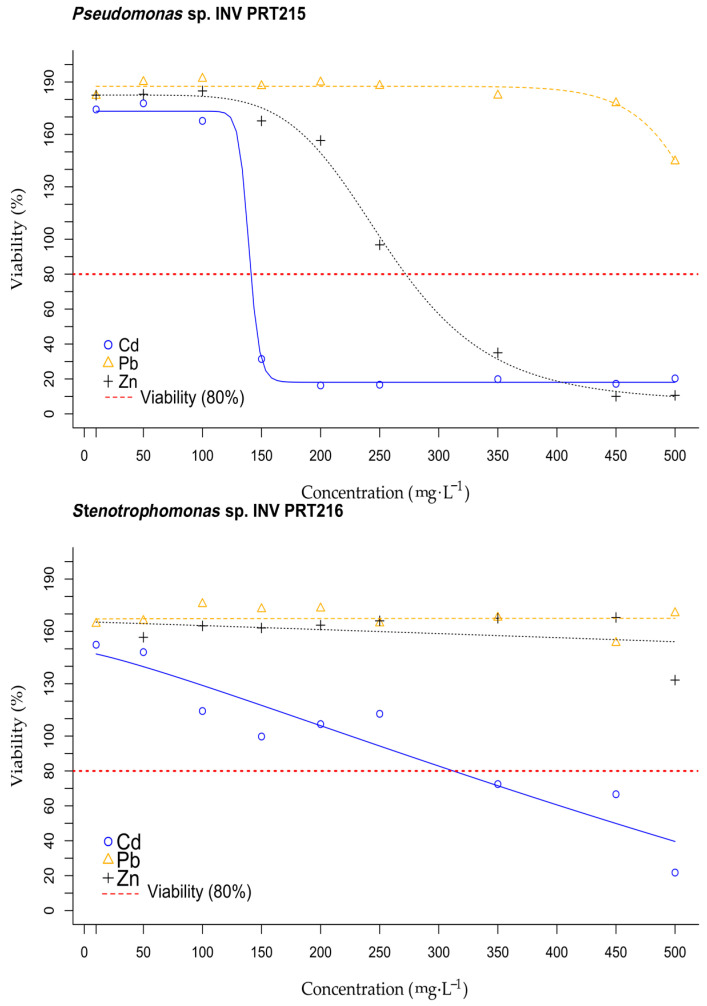
Viability percentages of bacteria INV PRT213, INV PRT215, and INV PRT216 at increasing concentrations of cadmium (blue), lead (yellow), and zinc (black). Concentrations ranged from 10 to 500 mg·L^−1^ and curves were adjusted using a nonlinear regression model of four parameters. Dots represent average values of triplicate samples and bars represent standard deviation 3.6. Growth curves of promising isolates *Enterobacter* sp. INV PRT213, *Pseudomonas* sp. INV PRT215, and *Stenotrophomonas* sp. INV PRT216 at different Pb^2+^ concentrations.

Figure 5 presents the results of viability trials conducted on bacteria INV PRT213, INV PRT215, and INV PRT216 at increasing concentrations of Cd^2+^, Pb^2+^, and Zn^2+^ (ranging from 10 to 500 mg·L^−1^). Average viability percentages greater than 100% indicated that microorganisms exposed to the heavy metals showed a higher cell metabolic activity than their respective controls (cultures without heavy metal). This is a phenomenon widely observed in bacteria particularly in heavy-metal tolerant strains, which have shown to increase their division rate as a response to environmental stress [15]. The results indicated that the viability of INV PRT216, INV PRT213 and INV PRT215 remained unaffected by lead exposure at the concentrations assessed in this study. Zinc exposure did not impact cell viability for INV PRT216, but the other two bacteria showed decreased viability after exposure to 250 mg·L^−1^ Zn^2+^. Cadmium had different effects on evaluated microorganisms: INV PRT213 displayed greater susceptibility to this metal, with viability being affected at approximately 35 mg·L^−1^, while INV PRT215 exhibited effects at around 125 mg·L^−1^ and INV PRT216 at 250 mg·L^−1^.

Among heavy metal pollutants, lead presence in several environments is of particular concern in Colombia and other countries due to its detrimental effects on human health and ecosystems, even at minimal concentrations exceeding 0.30 μg m^−3^ [50,51,52,53]. Consequently, Pb^2+^ was selected for further evaluation during growth curve construction and removal experiments.

### 3.6. Bacterial Growth Curves of Promising Isolates at Different Pb^2+^ Concentrations

**Figure 6:**
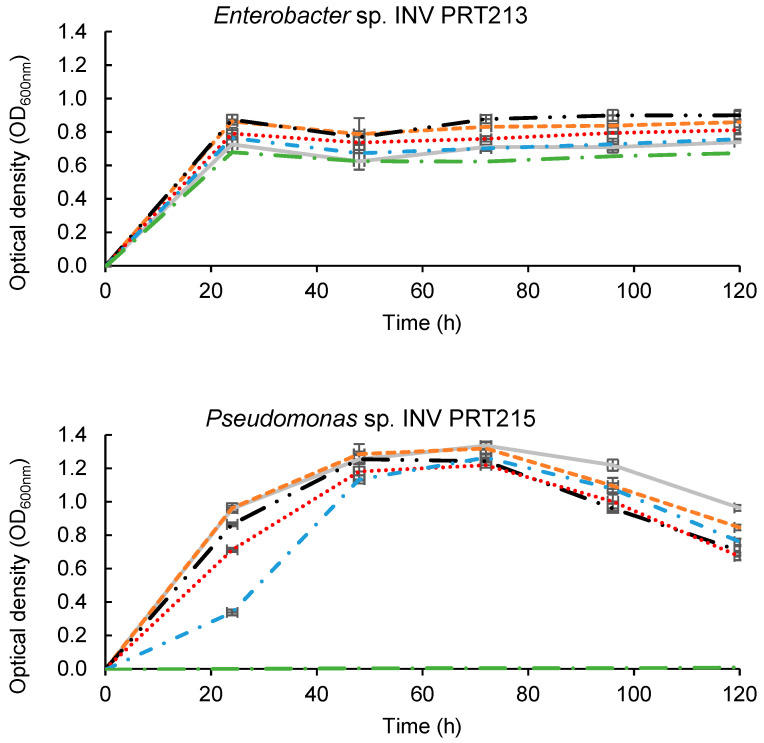

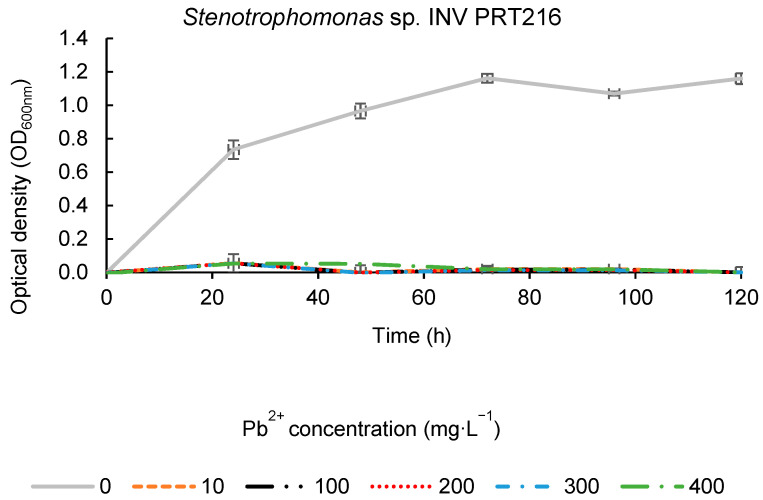
Growth curves of promising bacteria *Enterobacter* sp. INV PRT213, *Pseudomonas* sp. INV PRT215, and *Stenotrophomonas* sp. INV PRT216 at increasing lead concentrations. Dots represent average values of triplicate samples and bars represent standard deviation.

Figure 6 shows the growth curves of promising isolates at increasing Pb^2+^ concentrations. No lag phase was detected for the evaluated microorganisms, indicating their adaptation to the culture media. *Enterobacter* sp. INV PRT213 demonstrated robust growth at all evaluated concentrations, reaching its maximum OD_600nm_ after 24 h. A slight decrease in growth was observed between 48 and 72 h, after which it remained constant until 120 h of incubation (Figure 6). *Pseudomonas* sp. INV PRT215 exhibited adequate growth at lead concentrations up to 300 mg·L^−1^ Pb^2+^. It showed an exponential phase until ≈48 h of incubation, followed by a stationary phase, and a sharp decline at approximately 72 h (i.e., death phase) (Figure 6). A similar trend was observed in the control culture without lead (0 mg Pb^2+^·L^−1^).

On the other hand, it was evident that *Stenotrophomonas* sp. INV PRT216 was significantly affected by the presence of the metal, recording a maximum OD of 0.05 ± 0.09 at all Pb^2+^ concentrations. This represented an 18-fold reduction in OD compared to the control without metal (Figure 6). The intolerance to lead, as suggested by these results, seemed to contradict the findings obtained during tolerance trials and viability tests. However, it was hypothesized that cells present in the initial inoculum may have high affinity for the metal, effectively removing it from the growth media. As the stressor concentration decreased, it allowed the microorganism to divide, explaining the strong growth observed in agar plates and the positive viability results.

### 3.7. Evaluation of Pb^2+^ Removal Capacity of Promising Isolates Enterobacter sp. INV PRT213, Pseudomonas sp. INV PRT215, and Stenotrophomonas sp. INV PRT216

**Figure 7:**
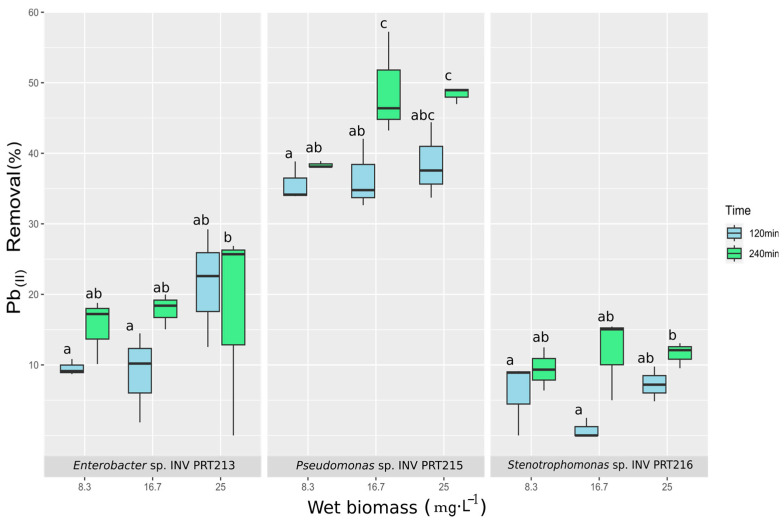
Average lead removal percentages + for bacteria *Enterobacter* sp. INV PRT213, *Pseudomonas* sp. INV PRT215, and *Stenotrophomonas* sp. INV PRT216. Removal experiments were performed at an initial Pb^2+^ concentration of 400 mg·L^−1^, 30 °C, 140 RPM, and an initial pH value of 5.0. Values represent differences between experimental runs and no inoculum controls. Statistical comparisons were made within columns. Means sharing a letter were not statistically different (Tukey test, *p* < 0.05).

Heavy-metal removal experiment results indicated that the wet biomass of *Enterobacter* sp. INV PRT213, *Pseudomonas* sp. INV PRT215, and *Stenotrophomonas* sp. INV PRT216 have different potential for treating lead -containing solutions (400 mg·L^−1^ Pb^2+^). Quantification of the total Pb via atomic absorption of the cell-free supernatants showed that *Pseudomonas* sp. INV PRT215 had the highest removal capacity among evaluated bacteria. (Figure 7). Average removal percentages ranged from 37 to 49%, reaching the maximal values at biomass concentrations ≥ 16.5 g·L^−1^ and a contact time of 240 min. Shorter contact times with this microorganism resulted in lower removal percentages of around 36%. Statistical analysis confirmed that removal efficiency was mediated by the biomass concentration (Tukey test, *p* < 0.05). A change in culture media coloration was observed when the biomass was exposed to the metal, i.e., from pale yellow to a darker color. An increase in culture pH was also detected, reaching final values close to 7.0 [54]. *Enterobacter* sp. INV PRT213 and *Stenotrophomonas* sp. INV PRT216 exhibited lower average removal percentages than *Pseudomonas* sp. INV PRT215. *Stenotrophomonas* sp. INV PRT216 removal results varied from 0 to 15%, with the maximum value achieved at a wet biomass dosage of 16.5 g·L^−1^ after 240 min of culture. As previously noted for the *Pseudomonas* strain, pH increased during culture to values around 7.0, but no changes in the LB medium’s coloration were observed. Viability results (Section 3.5) indicated that this microorganisms’ growth was largely affected by lead; therefore, we hypothesized that observed removal percentages can be due to Pb attachment to dead cells.

As for *Enterobacter* sp. INV PRT213, average removal percentages varied from 10 to 26%. The highest removal percentages were achieved at biomass concentrations of 25 g·L^−1^ regardless of contact time. No changes in the culture medium coloration were observed, and the final pH showed a slight increase reaching a value of around 6.0.

### 3.8. Analysis of Pseudomonas sp. INV PRT215 Biomass via Infrared Spectroscopy (FT-IR)

**Figure 8:**
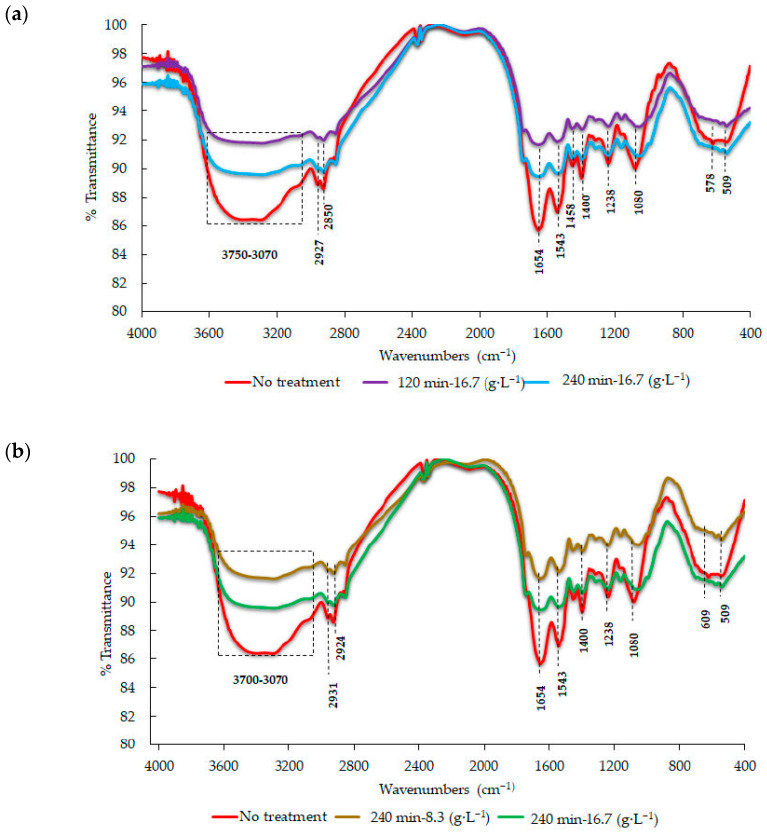
FT-IR analysis of *Pseudomonas* sp. INV PRT215 biomass in heavy-metal-free cultures and exposed to lead. Changes associated with the interaction between bacterial biomass of *Pseudomonas* sp. INV PRT215 and lead ions: (**a**) same amount of biomass, different times; (**b**) same contact time, different biomass concentration.

The IR spectra for the metal-free bacterial biomass (*Pseudomonas* sp. INV PRT215) as well as those obtained after Pb^2+^ removal trials are shown in Figure 8. The absorbance widebands for the extension vibrations of the hydroxyl and amine groups were located between 3433 and 3294 cm^−1^. The bands located between 2927 and 2850 cm^−1^ are symmetrical and asymmetrical extensions of the C-H bond attributed to alkyl chains [54]. There were also intense bands attributed to the stretching vibrations of carbonyl (C=O) of the amide functional groups at 1654 cm^−1^, as well as at 1543 cm^−1^ for carboxylates (COO-) [54]. On the other hand, the peaks located at 1238 cm^−1^ indicated vibrations associated with the possible presence of -SO_3_ groups. Finally, there were signs of the tension vibrations of phosphate groups at 1080 cm^−1^, as well as of those corresponding to the sulfur–oxygen bond (S-O) of organic sulphate groups at 709 cm^−1^ (Table 2) [54].

Close inspection of Figure 8 showed some differences in biomass spectra of *Pseudomonas* sp. INV PRT215 exposed to a Pb^2+^ solution. These changes were attributed to interactions of cellular components with the metal ion, which could be described as follows. (1) The carbonyl groups (C=O) of amides likely present in the cell membrane decreased considerably [54]. (2) The band located at 1543 cm^−1^, which corresponds to the carboxylates (COO-), exhibited a decrease in intensity, possibly due to the biosorption of the metal ion [54]. (3) The decrease in the transmittance percentage at 1080 cm^−1^ could be attributed to the interaction between the metal ion with phosphate groups [54]. (4) The wideband located in the 3433–3294 cm^−1^ range, corresponding to the hydroxyl and amine groups, experienced a change in intensity after the addition of the metal ion, possibly due to complexing between the metal and these functional groups [58]. It was also evidenced that, regardless of the time (Figure 8a) and biomass concentration (Figure 8b), the changes always took place within the same functional groups.

## 4. Discussion

The Chocó region in Colombia is considered a biodiversity hotspot located between the Caribbean and the Pacific Ocean. This area is geographically isolated from the Amazon basin by the Andes Mountain range, and it harbors one of the largest numbers of endemic species and abundant water and mineral resources. However, reports evaluating its microbial diversity are scarce.

Within this region there are three important national water bodies: the Atrato, San Juan, and Baudó Rivers [22]. Unfortunately, several reports have alerted the increasing presence of various serious contaminants in these rivers, including heavy metals, hydrocarbons, pesticides, and others. Heavy metal pollution is of particular concern as even small concentrations of these substances have shown to impact human health and disrupt metabolic processes throughout the food chain [22,59]. The introduction of heavy metals such as cadmium (Cd) and lead (Pb) has been primarily associated with the disposal of tailings from gold mining [60], while the presence of zinc (Zn) has been linked to runoff of superphosphate and other phosphorate fertilizers [61]. In this study, sediment samples were collected from the Atrato, San Juan, and Baudó rivers mouths and cultivable microorganisms were isolated from these remote and underexplored ecosystems. The isolated yeast and bacteria were assessed for their ability to tolerate heavy metals of interest (Cd, Pb and Zn), and promising organisms were further evaluated for their potential to remove lead from liquid solutions.

Based on the physicochemical parameters of the sampling sites, the salinity values correspond to brackish waters, indicating oligohaline zones due to the influence of the sea on the rivers [47]. The lower salinity values at some sampling points can be attributed to the influx of freshwater from the rivers, which was greatly influenced by the rainy season. The pH values of the surface water samples suggest a zone of freshwater influence, which tends to be more neutral compared to the expected ocean pH (around 8.1). However, in line with the quality criteria established by Colombian government agencies for the preservation flora and fauna [62], the pH levels at the sampling sites align with those found in marine and estuarine waters.

Classical microbiology approaches allowed for the isolation and characterization of microorganisms from the different sediment samples. The cultivable microorganisms included fungi and bacteria, while microalgae and other microorganisms were not isolated in this study. Despite using typical media for fungal isolation, such as B&K, malt extract agar, and RBC agar, the majority of the isolates were bacteria. Moreover, these media were supplemented with antibacterial molecules; therefore, bacterial growth indicated that there were antibiotic-resistant microorganisms in the sampled environments. These findings are consistent with numerous marine biodiversity studies that have reported a higher prevalence of cultivable bacteria compared to other microorganisms [63,64]. This is not unexpected, as there are still significant knowledge gaps regarding the appropriate culture of marine microorganisms, particularly of marine fungi development in laboratory settings [65,66,67]. Therefore, alternative approaches, like metagenomic analysis of the environmental DNA, could prove invaluable in elucidating the microbial diversity within the collected samples.

A greater number of cultivable strains, exhibiting different morphological characteristics were obtained from sampling points near mangrove zones, with isolate counts ranging from 39 to 55. This was in contrast to sampling points closer to the open sea (OBR. *n* = 32) or a beach (BSJM2, *n* = 34). Mangrove ecosystems, typically found along the coastal margins and brackish waters of coastal estuaries, provide complex nutrients (organic matter) and more favorable conditions for microbial colonization [68]. This favorable environment could explain the higher number of isolates from these areas.

Tolerance screening revealed that the majority of isolated microorganisms were capable of growing in at least one heavy metal at the levels assessed in this study (50–750 mg·L^−1^). Only five microorganisms did not exhibit colony development in any tolerance trials. It has been reported that gold mining occurs upstream of the Atrato, San Juan, and Baudó rivers, with wastewater influence downstream [22,31,32]. Therefore, it is possible that the presence of inorganic pollutants, such as heavy metals, in the collected sediments may have alter microbial metabolism and community structures [69], thus fostering the development of heavy metal-resistant strains in the selected sampling points.

The isolated microorganisms demonstrated a higher susceptibility to cadmium than to lead and zinc. Specifically, 64 microorganisms were completely inhibited by cadmium, while only nine were affected by all evaluated levels of Pb and Zn. Higher toxicity of cadmium compared to other heavy metals has been previously reported for several marine-derived microorganisms [70,71,72]. Authors argued that the ability of bacteria to tolerate different concentrations of heavy metals depends on many factors, including their taxonomic genus, metabolic abilities, growth conditions, and growth phase [73]. For instance, some studies have indicated that culture conditions, such as pH and salinity levels, play crucial roles in expressing cadmium resistance genes [74]. Metal-tolerant microorganisms can induce different genomic responses to avoid disrupting metabolic activity and prevent irreversible cell damage caused by heavy metals [75]. One of these defense mechanisms involves the production of protective mucilaginous substances in response to the metal presence, as widely described in the literature [48,76]. For instance, studies conducted in [76] reported the production of exopolysaccharides (EPS) by some creamy bacteria, which could chelate different metal ions (Pb, Cd, Cu, Zn, Ni, Co). Similarly, [77] showed that the bacterium *Paenibacillus jamilae* produced EPS with a strong affinity for complexing lead (228 mg g^−1^), effectively removing the pollutant from the aqueous solution. While the production of pigments and mucilaginous substances was observed in this study during screening trials, their exact role in heavy metal detoxification remains to be fully elucidated.

Molecular identification of selected promising isolates placed these microorganisms within the phylum Pseudomonadota (previously denoted as Proteobacteria), and they were assigned to three genera: *Stenotrophomonas*, *Enterobacter*, and *Pseudomonas*. Pseudomonadota has been described as the most common phylum in several sediment samples from marine environments [63,64]. Moreover, metagenomic analysis of sediment samples collected in the Colombian Caribbean region had also found Proteobacteria along with Bacteriodetes (Bacteroidota), Actinobacteria (Actinomycetota), and Firmicutes (Bacillota) as the dominant phyla, while *Pseudomonas* were the most common genera [78,79]. The genera *Enterobacter* and *Stenotrophomonas* have been documented in marine samples from around the world [63,80]. For instance, the genus *Enterobacter* has been isolated from marine water and sediment samples obtained from Cove Rock and Bonza Bay beach of the Eastern Cape Province, South Africa [80]. Similarly, the genus *Stenotrophomonas* has been reported in samples from the northeastern South China Sea [63]. Therefore, the findings of this study align with the global distribution of these microorganisms in marine environments.

The three microorganisms selected for further studies showed different growth trends when exposed to lead-containing solutions. These differences may be attributed to their distinct taxonomic classifications, which are directly associated to differences in metabolic capabilities [81]. Consequently, these bacteria showed different lead removal potentials, which could indicate different detoxification mechanisms for each bacterium. For example, the lead removal results obtained with the *Stenotrophomonas* strain (0–15%) and its corresponding growth curve data suggested that lead removal from the solution was a surface process, probably an adsorption process independent of metabolism: in this process, the metal was retained in some component of the dead biomass. These results contrast with those obtained by [82], who evaluated the tolerance of *Stenotrophomonas maltophilia* to different concentrations of Cd, Pb, Ag, Cu, and Ni ions, finding that, within 16 h, this microorganism could tolerate Pb(NO_3_)_2_ concentrations of up to 5 mM. Additionally, strains within this genus have been found to employ at least two mechanisms to overcome metal toxicity. These mechanisms involve the reduction of ions in their elemental state, rendering them non-toxic, and active detoxification processes that require an active metabolism [70,72,82].

Unlike the *Stenotrophomonas* sp. INV PRT216, *Enterobacter* sp. INV PRT213 and *Pseudomonas* sp. INV PRT215 showed higher removal capacities and active biomass division during the construction of growth curves. It was observed that lead removal was influenced by the interaction between biomass concentration and exposure time: as these two parameters increased, an increased removal percentage was observed. Therefore, these results were associated with a possible biosorption process. Biosorption mechanisms using living cells have been described as a two-stage process: an initial absorption independent of the metabolism that takes place after exposure, followed by a slower active absorption that does depend on active metabolism, generating an intracellular accumulation of metal ions [41]. Considering that short contact times were employed in this study, it is necessary to conduct longer exposure experiments that allow for a potentially higher removal percentage and active metal capture [83].

The bacterium *Pseudomonas* sp. INV PRT215 achieved the highest lead removal percentage (49%) in this study after 240 min of exposure, representing a removal efficiency of 0.29 mg Pb h^−1^ mgbiomass^−1^. These lead removal results are lower compared to those obtained with *Bacillus thioparans* strain U3 (1.88 mg Pb h^−1^ mgbiomass^−1^). However, they are comparable to those obtained with *Pseudomonas chengduensis* PPSS-4 (0.11 mg Pb h^−1^ mgbiomass^−1^) isolated from marine sediment samples from India [12], and they are at least 4-fold higher than those reported for gram-positive species such as *Lactobacillus plantarum* MF042018 (0.07 mg Pb h^−1^ mgbiomass^−1^) [43], and *Bacillus xiamenensis* PbRPSD202 (0.06 mg Pb h^−1^ mgbiomass^−1^) [41]. Gram-positive bacteria possess a thick peptidoglycan layer, which confers a negative charge on the cell wall and, therefore, a greater capacity to attract positively charged molecules such as Pb ions [84]. Even though *Pseudomonas* are Gram-negative bacteria and, consequently, they lack a heavily negative charge in their cell surface, other authors have reported several *Pseudomonas* species as an excellent heavy-metal biosorbents [54].

The FTIR analysis conducted on the biomass of the strain *Pseudomonas* sp. INV PRT215 evidenced possible interactions between Pb ions and functional groups in the cell, such as carboxylates, phosphates, and amines. It was observed on resulting spectra that transmittance decreased in biomass exposed to the metal compared to biomass from controls, indicating that the stretching of the bonds took place to a lesser extent when there was interaction with metals, thus reducing the intensity of the signals. Studies conducted by [54] suggested that the biomass of *Pseudomonas stutzeri* reduced the intensity of the functional groups after treatment with 300 mg·L^−1^ of Zn^2+^ and Pb^2+^. It has been reported that the ligands (functional groups) of the cell membrane with a negative load are useful for eliminating metals and other toxic compounds via electrostatic interactions [81]. Thus, the bonding sites with the greatest potential in the microorganisms cells are the carboxyl, amine, phosphate, sulphate, and hydroxyl groups [73], i.e., those recorded in this study during biomass FTIR analysis.

## 5. Conclusions

Classical microbiology culture methods allowed the isolation of several cultivable bacteria and fungi from sediment samples collected at underexplored tropical marine environments. Isolates exhibited various morphological and phenotypic characteristics, including pigmented, sporulated, filamentous, creamy, and mucilaginous microorganisms. Tolerance screening trials, focusing on three heavy metals—cadmium, lead, and zinc—revealed that the majority of the marine organisms exhibited some degree of tolerance to one or more of these metals at the evaluated concentration levels. Furthermore, the results indicated a more widespread sensitivity to cadmium than to lead and zinc among the evaluated marine organisms. Promising bacteria with high heavy-metal tolerance were identified through 16S rRNA gene sequencing, and they were found to belong to the phylum *Pseudomonadota*, specifically within the genera *Stenotrophomonas*, *Enterobacter*, and *Pseudomonas*. These assessed microorganisms displayed varying growth patterns when exposed to lead-containing solutions and demonstrated different potential for removing the metal from the aqueous phase. In particular, Gram-negative bacterium *Pseudomonas* sp. INV PRT215, isolated from San Juan River mouth, exhibited the highest lead removal efficiency surpassing the removal capacities of other marine bacteria, including some Gram-positive organisms. This study aimed to assess microbial diversity from underexplored marine environments and evaluate the heavy-metal tolerance of isolated microorganisms. The demonstrated growth and viability of multiple isolates in highly toxic metal solutions provided valuable insights for further studies aimed at enhancing the performance of marine microorganisms in heavy-metal remediation processes.

## Figures and Tables

**Table 1:** Sampling site location and physicochemical parameters of surface water.

Locality	Sampling Site	Sampling Site Abbreviation	Latitude	Longitude	Depth (m)	Temperature (°C)	Salinity (ppt)	pH
Atrato river mouth	Margarita Atrato river mouth	MARM	N 8°8′47.052″	W 76°50′8.337″	2.3	27.7	0.2	6.2
	Mangrove Paila Atrato river	MPAR	N 8°1′51″	W 76°50′51″	1.1	28.0	7.5	6.5
	Mangrove Roto Atrato river	MRAR	N 8°12′42.351″	W 76°56′33.552″	3.2	27.6	0.0	6.2
Baudó river mouth	Mangrove via Usaraga Baudó river	MVUBR	N 4°56′38.4″	W 77°21′48.6″	0.5	27.1	7.2	6.2
	Out to sea Baudó river	OBR	N 4°56′19.9″	W 77°22′55.1″	7.6	29.7	6.5	6.4
San Juan river mouth	Mangrove Choncho San Juan river two	MCHSJ2	N 4°2′56.2″	W 77°27′03.1″	4.0	25.9	0.3	6.3
	Mangrove San Juan river two	MSJ2	N 4°2′34.5″	W 77°26′05.1″	4.0	26.2	3.1	6.4
	Beach San Juan river mouth two	BSJM2	N 4°1′31.2″	W 77°26′24.1″	4.5	25.6	1.5	6.9

**Table 2:** Changes in the functional groups present in the cell membranes of strains treated with and without Pb^2+^.

Wavenumber (cm^−1^)	Group Functional	Wavenumber (cm^−1^) after Lead Removal	References
3433 and 3294	Hydroxyl groups	3263–3140	[54,55,56,57]
1080	Phosphate	1118	
1780–1640	(C=O) of amides	1658–1539	
1543	Carboxylate (COO-)	1519	
1238	SO_3_	1045	

## Data Availability

The data presented in this study are available in supplementary material.

## Supplementary Materials

The following supporting information can be downloaded at: https://www.mdpi.com/article/10.3390/microorganisms11112708/s1. Table S1. Culture media for microbial isolation. Table S2. Cadmium (Cd^2+^), Lead (Pb^2+^), and Zinc (Zn^2+^) tolerance screening of 276 microorganisms. A microorganism was catalogued as tolerant (+) if growth was observed after 24–48 h of culture; not tolerant (−) if growth was inhibited entirely around wells, or sensitive (±) if some colonies were formed (36). Sampling sites: Atrato river mouth (MPAR, MARM, MRAR), Baudó river mouth (OBR, MVUBR), and San Juan river mouth (MCHSJ2, BSJM2, MSJ2). Table S3. Cadmium (Cd^2+^), Lead (Pb^2+^), and Zinc (Zn^2+^) tolerance screening trials at increased concentrations of Pb^2+^, Cd^2+^ and Zn^2+^ ions of eleven microorganisms. A microorganism was catalogued as tolerant (+) if growth was observed after 24–48 h of culture; not tolerant (−) if growth was inhibited entirely around wells, or sensitive (±) if some colonies were formed (36). Table S4. Results obtained by comparing the 16S rRNA sequences to those in the Reference RNA Sequences database of the NCBI. Figure S1. Phenotypic characteristics of some isolated colonies. Figure S2. Photographic record of pigments and substances excreted by bacterium 76955 under Cd^2+^ and Zn^2+^ stress conditions. Figure S3. Number of strains tolerant to Cd^2+^, Pb^2+^, and Zn^2+^ ions by sampling site. Sampling sites: Atrato river mouth (MPAR, MARM, MRAR), Baudó river mouth (OBR, MVUBR), and San Juan river mouth (MCHSJ2, MSJ2, BSJM2).
