# Supplementary material for: Heavy Metal Tolerance of Microorganisms Isolated from Coastal Marine Sediments and Their Lead Removal Potential
Source: Microorganisms. 2023 Nov 4;11(11):2708. doi: 10.3390/microorganisms11112708 (PMC10673411; doi:10.3390/microorganisms11112708)
Supplement: Supplementary file 1 [file microorganisms-11-02708-s001.zip › microorganisms-2645829-supplementary.pdf]

Supplementary material: Heavy Metal Tolerance of Microorganisms isolated from Coastal Marine Sediments and Their Lead Removal Potential

Katleen L. Alvarado-Campo [1,†]., Marynes Quintero [1,†]., Bernarda Cuadrado-Cano [2],
Manuela Montoya-Giraldo [1,‡], Elver Luis Otero-Tejada[1], Lina Blandón[1], Olga Sánchez[3], Ana Zuleta-Correa[1,*], and Javier Gómez-León [1]

[1] Marine Bioprospecting Line, Evaluation and Use of Marine and Coastal Resources Program–VAR, Marine and Coastal Research Institute–INVEMAR, Santa Marta 470006, Magdalena, Colombia; katleen.alvarado@invemar.org.co (K.L.A.-C.); marynes.quintero@invemar.org.co (M.Q.); elverotero@sophicol.org (E.L.O.-T.); lmblando@unal.edu.co (L.B.); javier.gomez@invemar.org.co (J.G.-L.)

[2] Master's Program in Microbiology, College of Medicine, Universidad de Cartagena, Cartagena de Indias 130014, Bolívar, Colombia; bcuadradoc@unicartagena.edu.co

[3] Department of Genetics and Microbiology, Faculty of Biosciences, Universitat Autònoma de Barcelona, Bellaterra 08193, Spain; olga.sanchez@uab.cat

[*] Correspondence: ana.zuleta@invemar.org.co

[†] These authors contributed equally to this work.

[‡] Current address: Department of Biological Sciences, University at Albany, State University of New York, Albany 12222, NY, USA.

Supplementary material Table S1. Culture media for microbial isolation

Culture media	Components (g.L^{-1})		pH	Processing method
Starch casein agar (SCA)	Soluble starch	10	7.2 ± 0.2	Direct seeding of no-pre-treatment sediments, phenol pre-treatment and selective pressure method ($HgCl_2$)
	Casein	0.3		
	Sodium nitrate	2.0		
	Sodium chloride	2.0		
	Magnesium sulphate	0.05		
	Calcium carbonate	0.02		
	Iron sulphate	0.01		
	Seawater: distilled water	60:40		
	Agar	18		
Either Boyd and Kohlmeyer (B&K) medium	Yeast extract	1.0	7.0 ± 0.2	Direct seeding of no-pre-treatment sediments and selective pressure method ($HgCl_2$)
	Peptone	2.0		
	Glucose	10		
	Seawater: distilled water	1:1		
	Streptomycin antibiotic	0.1		
	Agar	18		
Malt Extract agar	Malt extract	30.0	5.4 ± 0.2	Direct seeding of no-pre-treatment

Culture media	Components ($g.L^{-1}$)		pH	Processing method
	Peptone	5.0		sediments and selective pressure method ($HgCl_2$)
	Streptomycin antibiotic	0.1		
	Agar	15		
International *Streptomyces* project 2 (ISP2) medium	Yeast extract	4.0	7.2 ± 0.2	Direct seeding of no-pre-treatment sediments and selective pressure
	Malt extract	10		
	Glucose	4.0		
	Sodium chloride	10		
	Agar	18		
International *Streptomyces* project 4 (ISP4) medium	Soluble starch	10	7.2 ± 0.2	Phenol pre-treatment and selective pressure method ($HgCl_2$)
	Dibasic potassium phosphate	1.0		
	Magnesium sulphate heptahydrate	1.0		
	Sodium chloride	10		
	Ammonium sulphate	2.0		
	Calcium carbonate	2.0		
	Iron sulphate heptahydrate	0.001		
	Manganese (II) chloride tetrahydrate	0.001		
	Zinc sulphate heptahydrate	0.001		
	Agar	18		
International *Streptomyces* project 3 (ISP3) medium	White Oats	20	7.3 ± 0.2	Selective pressure method ($HgCl_2$)
	Iron sulphate heptahydrate	0.001		
	Manganese (II) chloride tetrahydrate	0.001		
	Zinc sulphate heptahydrate	0.001		
	Agar	18		
Marine agar Difco™ (MA)	Commercial preparation			Direct seeding of no-pre-treatment sediments, phenol pre-treatment and selective pressure method ($HgCl_2$)
Rose Bengal Chloramphenicol agar (RBC) Merck®	Commercial preparation			Direct seeding of no-pre-treatment sediments and selective pressure method ($HgCl_2$)
Tryptic soy agar (TSA) Scharlau Scharlab, S.L	Commercial preparation			Direct seeding of no-pre-treatment sediments

Culture media	Components (g.L^{-1})	pH	Processing method
Actinobacteria agar (AA) Merck®	Commercial preparation		Direct seeding of no-pre-treatment sediments

Supplementary material Table S2. Cadmium (Cd^{2+}), Lead (Pb^{2+}), and Zinc (Zn^{2+}) tolerance screening of 276 microorganisms. A microorganism was catalogued as tolerant (+) if growth was observed after 24-48 h of culture; not tolerant (-) if growth was inhibited entirely around wells, or sensitive (±) if some colonies were formed (36). Sampling sites: Atrato river mouth (MPAR, MARM, MRAR), Baudó river mouth (OBR, MVUBR), and San Juan river mouth (MCHSJ2, BSJM2, MSJ2).

Sampling site	Consecutive number MHNMC	Type of microorganisms	Tolerance screening on Luria Bertani agar (LB)											
			Cadmium (Cd) mg.L^{-1}				Lead (Pb) mg.L^{-1}				Zinc (Zn) mg.L^{-1}			
			50	150	250	350	50	150	250	350	50	150	250	350
MPAR	76844	Bacteria	-	-	-	-	+	+	+	+	+	-	-	-
MPAR	76845	Bacteria	-	-	-	-	+	+	+	+	+	+	+	+
MPAR	76846	Bacteria	-	-	-	-	+	-	-	-	+	+	-	-
MPAR	76847	Bacteria	-	-	-	-	+	+	+	+	+	+	+	+
MPAR	76848	Bacteria	+	+	+	-	+	+	+	+	+	+	+	+
MPAR	76849	Bacteria	+	+	+	+	+	+	+	+	+	+	+	+
MPAR	76850	Bacteria	-	-	-	-	+	+	+	+	±	±	±	-
MPAR	76851	Bacteria	-	-	-	-	-	-	-	-	-	-	-	-
MPAR	76852	Bacteria	-	-	-	-	-	-	-	-	-	-	-	-
MPAR	76853	Bacteria	±	-	-	-	+	+	+	+	+	+	+	+
MPAR	76854	Bacteria	±	±	-	-	+	+	+	+	+	+	+	+
MPAR	76855	Bacteria	-	-	-	-	+	+	+	+	+	-	-	-
MPAR	76856	Bacteria	-	-	-	-	+	+	+	+	+	+	+	+
MPAR	76857	Bacteria	-	-	-	-	+	-	-	-	+	+	+	-
MRAR	76858	Bacteria	+	+	+	+	+	+	+	+	+	+	+	+
MRAR	76859	Bacteria	+	+	+	+	+	+	+	+	+	+	+	+
MRAR	76860	Bacteria	+	+	+	+	±	±	±	±	+	+	+	±
MRAR	76861	Bacteria	+	+	+	+	+	+	+	+	+	+	+	+
MRAR	76862	Bacteria	+	+	+	+	+	+	+	+	+	+	+	+
MRAR	76863	Bacteria	-	-	-	-	+	+	+	+	+	+	+	-
MRAR	76864	Bacteria	-	-	-	-	+	+	+	+	+	+	+	+
MRAR	76865	Bacteria	+	+	+	+	+	+	+	+	+	+	+	+
MRAR	76866	Bacteria	+	+	+	+	+	+	+	+	+	+	+	+
MRAR	76867	Bacteria	+	+	-	-	+	+	+	+	+	+	+	+
MARM	76868	Bacteria	+	+	±	±	+	+	+	+	+	+	+	+
MARM	76869	Bacteria	+	+	+	+	+	+	+	+	+	+	+	+

Sampling site	Consecutive number MHNMC	Type of microorganisms	Tolerance screening on Luria Bertani agar (LB)											
			Cadmium (Cd) $mg.L^{-1}$				Lead (Pb) $mg.L^{-1}$				Zinc (Zn) $mg.L^{-1}$			
			**50**	**150**	**250**	**350**	**50**	**150**	**250**	**350**	**50**	**150**	**250**	**350**
MARM	76870	Bacteria	+	+	+	±	+	+	+	+	+	+	+	±
MARM	76871	Bacteria	±	-	-	-	+	+	+	+	+	+	±	-
MARM	76872	Bacteria	+	+	+	+	+	+	+	+	+	+	+	+
MARM	76873	Bacteria	+	+	+	+	+	+	+	+	+	+	+	+
MARM	76874	Bacteria	+	+	+	+	+	+	+	+	+	+	+	+
MARM	76875	Bacteria	+	+	-	-	+	+	+	+	+	+	+	+
MARM	76876	Bacteria	±	-	-	-	+	±	-	-	+	+	±	±
MARM	76877	Bacteria	+	+	+	+	+	+	-	-	-	-	-	-
MARM	76878	Bacteria	+	+	+	±	+	+	+	+	+	+	+	+
MARM	76879	Bacteria	+	+	+	+	+	+	+	+	+	+	+	+
MARM	76880	Bacteria	+	+	+	+	+	+	+	+	+	+	+	+
MARM	76881	Bacteria	+	+	+	+	+	+	+	+	+	+	+	+
MARM	76882	Bacteria	+	+	+	+	+	+	+	+	+	+	+	+
MARM	76883	Bacteria	+	+	+	+	+	+	+	+	+	+	+	+
MARM	76884	Bacteria	+	+	-	-	+	+	+	+	+	+	+	+
MARM	76885	Bacteria	-	-	-	-	+	+	+	+	+	-	-	-
MARM	76886	Bacteria	+	+	+	+	+	+	+	+	+	+	+	+
MARM	76887	Bacteria	-	-	-	-	+	+	+	+	±	±	±	±
MARM	76888	Bacteria	-	-	-	-	+	+	+	+	±	-	-	-
MARM	77109	yeast	+	+	+	+	+	+	+	+	+	+	+	+
MARM	77111	yeast	+	+	±	±	+	+	+	+	+	+	+	+
MRAR	77115	yeast	+	-	-	-	+	+	+	+	+	+	+	+
MRAR	77116	yeast	+	-	-	-	+	+	+	+	+	+	+	+
MRAR	77118	yeast	+	-	-	-	+	+	+	+	+	+	+	+
MPAR	77124	yeast	-	-	-	-	+	+	+	+	+	+	+	+
MRAR	77127	yeast	-	-	-	-	+	+	+	+	+	+	+	-
MRAR	77129	yeast	+	-	-	-	+	+	+	+	+	+	+	+
MRAR	76891	Bacteria	+	+	+	+	+	+	+	+	+	+	+	+
MRAR	76892	Bacteria	±	±	-	-	+	+	±	±	+	+	-	-
MRAR	76893	Bacteria	+	+	+	+	+	+	+	+	+	+	+	+
MRAR	76894	Bacteria	+	+	+	+	+	+	+	+	+	+	+	+
MPAR	76895	Bacteria	+	+	+	+	+	+	+	+	+	+	+	+
MPAR	76896	Bacteria	+	±	-	-	+	+	+	+	+	+	+	+
MPAR	76897	Bacteria	+	±	±	-	+	+	+	+	+	+	+	+
MPAR	76898	Bacteria	-	-	-	-	+	+	+	+	+	+	+	+
MPAR	76899	Bacteria	+	+	+	+	+	+	+	+	+	+	+	+
MPAR	76900	Bacteria	-	-	-	-	+	+	+	+	+	+	+	+

Sampling site	Consecutive number MHNMC	Type of microorganisms	Tolerance screening on Luria Bertani agar (LB)											
			Cadmium (Cd) mg.L^{-1}				Lead (Pb) mg.L^{-1}				Zinc (Zn) mg.L^{-1}			
			50	150	250	350	50	150	250	350	50	150	250	350
MPAR	76901	Bacteria	-	-	-	-	-	-	-	-	±	-	-	-
MPAR	76902	Bacteria	+	+	+	-	+	+	+	+	+	+	+	+
MPAR	76903	Bacteria	+	+	+	+	+	+	+	+	+	+	+	+
MARM	76905	Bacteria	+	+	+	+	+	+	+	+	+	+	+	+
MARM	76906	Bacteria	+	+	+	+	+	+	+	+	+	+	+	+
MARM	76907	Bacteria	-	-	-	-	+	+	+	+	+	+	+	-
MRAR	76908	Bacteria	-	-	-	-	+	+	+	+	+	±	-	-
MARM	76909	Bacteria	+	+	+	+	+	+	+	+	+	+	+	+
MARM	76910	Bacteria	+	+	+	+	+	+	+	+	+	+	+	+
MARM	76911	Bacteria	+	+	+	+	+	+	+	+	+	+	+	+
MARM	76912	Bacteria	±	-	-	-	+	+	+	+	+	+	±	±
MARM	76913	Bacteria	±	±	±	±	+	+	+	+	+	±	±	±
MARM	76914	Bacteria	-	-	-	-	+	+	+	+	+	+	±	±
MRAR	76916	Bacteria	+	+	+	+	+	+	+	+	+	+	+	+
OBR	76917	Bacteria	-	-	-	-	+	±	±	±	+	+	-	-
OBR	76918	Bacteria	+	+	+	+	+	+	+	+	+	+	+	+
OBR	76919	Bacteria	-	-	-	-	+	+	+	+	+	+	±	±
OBR	76920	Bacteria	-	-	-	-	+	+	+	+	+	+	+	+
OBR	76921	Bacteria	+	+	+	+	+	+	+	+	+	+	+	+
OBR	76922	Bacteria	±	±	±	±	+	+	+	+	+	±	±	±
OBR	76923	Bacteria	-	-	-	-	+	+	+	+	+	+	-	-
OBR	76924	Bacteria	+	-	-	-	+	+	+	+	+	+	+	+
OBR	76925	Bacteria	+	+	+	+	+	+	+	+	+	+	+	+
OBR	76926	Bacteria	+	±	-	-	+	+	+	+	+	+	+	+
OBR	76927	Bacteria	-	-	-	-	+	+	+	+	+	-	-	-
OBR	76928	Bacteria	±	-	-	-	+	+	+	+	+	+	+	±
OBR	76929	Bacteria	+	+	+	+	+	+	+	+	+	+	+	+
OBR	76930	Bacteria	+	±	±	-	+	+	+	+	+	+	+	+
MVUBR	76931	Bacteria	+	+	+	+	+	+	+	+	+	+	+	+
MVUBR	76932	Bacteria	-	-	-	-	+	+	+	+	+	+	+	+
MVUBR	76933	Bacteria	-	-	-	-	+	+	+	+	-	-	-	-
MVUBR	76934	Bacteria	+	±	±	±	+	+	+	+	+	+	+	+
MVUBR	76935	Bacteria	+	±	-	-	+	+	+	+	+	+	+	-
MVUBR	76936	Bacteria	-	-	-	-	+	+	+	+	+	+	+	+
MVUBR	76937	Bacteria	+	+	+	+	+	+	+	+	+	+	+	+
MVUBR	76938	Bacteria	+	-	-	-	+	+	+	+	+	+	+	+
MVUBR	76939	Bacteria	-	-	-	-	+	+	+	+	+	+	+	+

Sampling site	Consecutive number MHNMC	Type of microorganisms	Tolerance screening on Luria Bertani agar (LB)											
			Cadmium (Cd) mg.L^{-1}				Lead (Pb) mg.L^{-1}				Zinc (Zn) mg.L^{-1}			
			50	150	250	350	50	150	250	350	50	150	250	350
MVUBR	76940	Bacteria	-	-	-	-	+	+	+	+	+	+	+	+
MVUBR	76941	Bacteria	-	-	-	-	+	+	+	+	+	+	-	-
MVUBR	76942	Bacteria	+	+	+	+	+	+	+	+	+	+	+	+
MVUBR	76943	Bacteria	+	+	+	+	+	+	+	+	+	+	+	+
MVUBR	76944	Bacteria	±	±	±	±	±	±	±	±	+	+	+	+
MVUBR	76945	Bacteria	+	+	+	+	+	+	+	+	+	+	+	+
MVUBR	76946	Bacteria	+	±	-	-	+	+	+	+	+	+	+	+
MVUBR	76947	Bacteria	+	+	+	+	+	+	+	+	+	+	+	-
MVUBR	76948	Bacteria	+	+	+	+	+	+	+	+	+	+	+	+
MVUBR	76949	Bacteria	±	-	-	-	+	+	+	+	+	-	-	-
MVUBR	76950	Bacteria	+	-	-	-	+	+	+	+	+	+	+	-
MVUBR	76951	Bacteria	+	+	+	+	+	+	+	+	+	+	+	+
MVUBR	76952	Bacteria	+	+	+	+	+	+	+	+	+	+	+	+
MVUBR	76953	Bacteria	-	-	-	-	+	+	+	+	±	±	-	-
MVUBR	76954	Bacteria	±	-	-	-	+	+	+	+	+	+	+	+
OBR	77137	Bacteria	+	+	+	+	+	+	+	+	+	+	+	+
OBR	INV PRT216*	Bacteria	+	+	+	+	+	+	+	+	+	+	+	+
MVUBR	77141	Bacteria	+	+	+	+	+	+	+	+	+	+	+	+
MCHSJ2	76955	Bacteria	+	+	+	+	+	+	+	+	+	+	+	+
MCHSJ2	76956	Bacteria	+	+	+	+	+	+	+	+	+	+	+	+
MCHSJ2	76957	Bacteria	-	-	-	-	+	+	+	+	+	±	-	-
MCHSJ2	76958	Bacteria	±	-	-	-	+	+	+	+	+	+	±	-
MCHSJ2	76959	Bacteria	-	-	-	-	+	+	+	+	+	+	±	-
MCHSJ2	76960	Bacteria	+	+	±	±	-	+	+	+	+	+	+	+
MCHSJ2	76961	Bacteria	+	±	-	-	±	±	±	±	+	±	±	-
MCHSJ2	76962	Bacteria	±	-	-	-	+	+	+	+	+	+	±	-
MCHSJ2	76963	Bacteria	-	-	-	-	+	+	+	+	+	-	-	-
MCHSJ2	76964	Bacteria	+	-	-	-	+	+	+	+	+	+	+	+
MCHSJ2	76965	Bacteria	+	+	+	+	+	+	+	+	+	+	+	+
MCHSJ2	76966	Bacteria	-	-	-	-	+	+	+	+	+	-	-	-
MCHSJ2	76967	Bacteria	+	+	+	-	+	+	+	+	+	+	+	+
MCHSJ2	76968	Bacteria	+	-	-	-	+	+	+	+	+	+	-	-
MCHSJ2	76970	Bacteria	-	-	-	-	+	+	+	+	±	±	±	±
MCHSJ2	76971	Bacteria	-	-	-	-	+	+	+	±	+	+	+	+
MCHSJ2	76972	Bacteria	+	+	+	+	+	+	+	+	+	+	+	+
MCHSJ2	76973	Bacteria	+	+	+	+	+	+	+	+	+	+	+	+
MCHSJ2	76974	Bacteria	+	+	+	+	+	+	+	+	+	+	+	+

Sampling site	Consecutive number MHNMC	Type of microorganisms	Tolerance screening on Luria Bertani agar (LB)											
			Cadmium (Cd) mg.L^{-1}				Lead (Pb) mg.L^{-1}				Zinc (Zn) mg.L^{-1}			
			50	150	250	350	50	150	250	350	50	150	250	350
MCHSJ2	76975	Bacteria	-	-	-	-	-	-	-	-	-	-	-	-
MCHSJ2	76976	Bacteria	±	±	±	±	+	+	+	+	+	+	+	±
MCHSJ2	76977	Bacteria	+	+	+	-	+	+	+	+	+	+	+	+
MCHSJ2	76978	Bacteria	+	+	+	-	+	+	+	+	+	+	+	+
MCHSJ2	76979	Bacteria	±	±	±	±	±	±	±	±	+	+	+	+
MCHSJ2	76980	Bacteria	+	+	+	+	+	+	+	+	+	-	-	-
MSJ2	76981	yeast	±	±	-	-	+	+	+	+	+	+	+	+
MSJ2	76982	Bacteria	+	±	±	-	+	+	+	+	+	+	+	+
MSJ2	76983	Bacteria	+	-	-	-	+	+	+	+	+	+	+	+
MSJ2	76984	Bacteria	-	-	-	-	+	+	+	+	+	+	+	+
MSJ2	76985	Bacteria	-	-	-	-	+	+	+	+	+	+	+	-
MSJ2	76986	Bacteria	-	-	-	-	+	+	+	+	+	+	+	+
MSJ2	76987	Bacteria	+	+	+	+	+	+	+	+	+	+	+	+
MSJ2	76988	Bacteria	+	+	±	±	+	+	±	±	+	+	+	+
MSJ2	76989	Bacteria	-	-	-	-	+	+	+	+	+	+	+	-
MSJ2	76991	Bacteria	+	+	+	-	+	+	+	+	+	+	+	+
MSJ2	76992	Bacteria	+	±	-	-	+	+	+	+	+	+	+	+
MSJ2	76993	Bacteria	-	-	-	-	+	+	+	+	+	+	+	+
MSJ2	76994	Bacteria	±	-	-	-	+	+	+	+	+	+	+	-
MSJ2	76995	Bacteria	-	-	-	-	+	+	+	+	+	+	+	+
BSJM2	76996	Bacteria	-	-	-	-	+	+	+	+	-	-	-	-
BSJM2	76997	Bacteria	-	-	-	-	-	-	-	-	-	-	-	-
BSJM2	76998	Bacteria	-	-	-	-	+	+	+	±	+	+	±	±
BSJM2	76999	Bacteria	+	+	±	±	+	+	+	+	+	+	+	±
BSJM2	77000	Bacteria	±	±	±	±	+	+	-	-	-	-	-	-
BSJM2	INV PRT211*	Bacteria	+	+	+	+	+	+	+	+	+	+	+	+
BSJM2	77002	Bacteria	+	+	+	+	+	+	+	+	+	+	+	+
BSJM2	77003	Bacteria	+	+	-	-	+	+	+	+	+	+	+	+
BSJM2	77004	Bacteria	+	+	-	-	+	+	+	+	+	+	+	+
BSJM2	77005	Bacteria	-	-	-	-	+	+	+	+	+	+	+	±
MCHSJ2	77144	yeast	-	-	-	-	+	+	+	+	+	+	+	+
MCHSJ2	77145	yeast	-	-	-	-	+	+	+	+	+	+	+	+
MCHSJ2	77146	yeast	±	-	-	-	+	+	+	+	+	+	±	-
MCHSJ2	77149	yeast	-	-	-	-	-	-	-	-	+	+	+	+
MCHSJ2	77152	Bacteria	±	-	-	-	+	+	+	+	±	±	±	±
BSJM2	77153	Bacteria	+	+	+	+	+	+	+	+	+	+	+	+
BSJM2	77154	yeast	+	±	±	±	+	+	+	+	+	+	+	+

Sampling site	Consecutive number MHNMC	Type of microorganisms	Tolerance screening on Luria Bertani agar (LB)											
			Cadmium (Cd) mg.L^{-1}				Lead (Pb) mg.L^{-1}				Zinc (Zn) mg.L^{-1}			
			50	150	250	350	50	150	250	350	50	150	250	350
MCHSJ2	77156	yeast	+	+	+	+	+	+	+	+	+	+	+	+
MCHSJ2	77157	yeast	-	-	-	-	+	+	+	+	+	+	+	+
MCHSJ2	77158	yeast	-	-	-	-	+	+	+	+	+	+	+	+
MCHSJ2	77161	yeast	±	±	±	±	+	+	+	+	+	+	+	+
MSJ2	77161	yeast	+	+	+	+	+	+	+	+	+	+	+	+
MSJ2	77165	Bacteria	+	+	+	-	+	+	+	+	+	+	+	+
MSJ2	77166	yeast	-	-	-	-	+	+	+	+	+	+	+	+
MSJ2	77169	Bacteria	+	+	+	+	+	+	+	+	+	+	+	+
BSJM2	77006	Bacteria	+	+	+	+	+	+	+	+	+	+	+	+
BSJM2	77177	Bacteria	±	±	±	±	+	+	+	+	+	+	+	±
BSJM2	77179	yeast	+	+	+	+	+	+	+	+	+	+	+	+
MSJ2	77008	Bacteria	+	+	+	+	+	+	+	+	+	+	+	+
MSJ2	77009	Bacteria	+	±	±	±	+	+	+	+	+	+	+	+
MSJ2	77010	Bacteria	+	+	+	+	+	+	+	+	+	+	+	-
MSJ2	77011	Bacteria	+	+	+	+	+	+	+	+	+	+	+	+
MSJ2	77012	Bacteria	-	-	-	-	+	+	+	+	+	+	+	-
MSJ2	77013	Bacteria	±	±	±	±	+	+	+	+	+	+	+	+
MSJ2	77014	Bacteria	+	+	+	+	+	+	+	+	+	+	+	+
MSJ2	77015	Bacteria	±	±	±	±	+	+	+	+	+	+	+	+
MSJ2	77016	Bacteria	-	-	-	-	+	+	+	+	+	+	+	+
MSJ2	77017	Bacteria	+	+	±	-	+	+	+	+	+	+	+	+
BSJM2	77018	Bacteria	+	+	+	+	+	+	+	+	+	+	+	+
BSJM2	77019	Bacteria	+	+	+	+	+	+	+	+	+	+	+	+
BSJM2	77020	Bacteria	+	+	+	+	+	+	+	+	+	+	+	+
BSJM2	77021	Bacteria	+	+	+	+	+	+	+	+	+	+	+	+
BSJM2	77022	Bacteria	+	+	±	-	+	+	-	-	+	+	+	+
MSJ2	77023	Bacteria	+	+	+	+	+	+	+	+	+	+	+	+
MSJ2	77024	Bacteria	+	±	-	-	+	+	+	+	+	+	+	+
BSJM2	77027	Bacteria	+	+	+	+	+	+	+	+	+	+	+	+
BSJM2	77028	Bacteria	+	+	+	+	+	+	+	+	+	+	+	+
MRAR	77029	Bacteria	+	+	+	+	+	+	+	+	+	+	+	+
MRAR	INV PRT212*	Bacteria	+	+	+	+	+	+	+	+	+	+	+	+
MRAR	77031	Bacteria	+	+	+	+	+	+	+	+	+	+	+	+
MRAR	77032	Bacteria	+	+	+	±	+	+	+	+	+	+	+	+
MRAR	77033	Bacteria	-	-	-	-	+	+	+	+	+	+	+	±
MRAR	INV PRT213*	Bacteria	+	+	+	+	+	+	+	+	+	+	+	+

Sampling site	Consecutive number MHNMC	Type of microorganisms	Tolerance screening on Luria Bertani agar (LB)											
			Cadmium (Cd) mg.L^{-1}				Lead (Pb) mg.L^{-1}				Zinc (Zn) mg.L^{-1}			
			50	150	250	350	50	150	250	350	50	150	250	350
MRAR	77035	Bacteria	+	+	+	+	+	+	+	+	+	+	+	+
MRAR	77036	Bacteria	+	+	+	+	+	+	+	+	+	+	+	+
MRAR	77037	Bacteria	+	+	+	+	+	+	+	+	+	+	+	+
MPAR	77038	Bacteria	+	-	-	-	+	+	+	+	+	+	+	+
MPAR	77039	Bacteria	±	±	-	-	+	+	+	+	±	±	±	±
MPAR	77040	Bacteria	±	±	±	±	+	+	+	+	+	+	+	+
MPAR	77041	Bacteria	+	+	+	+	+	+	+	+	+	+	+	+
MPAR	77042	Bacteria	+	+	±	-	+	+	+	+	+	+	+	+
MPAR	77044	Bacteria	+	-	-	-	+	+	+	+	+	+	+	+
MPAR	77045	Bacteria	+	+	+	+	+	+	+	+	+	+	+	+
MCHSJ2	77046	Bacteria	+	+	+	±	±	±	±	-	+	+	+	+
MCHSJ2	77047	Bacteria	+	+	+	-	-	-	-	-	+	+	+	+
MCHSJ2	77048	Bacteria	+	+	+	+	+	+	+	+	+	+	+	+
MCHSJ2	77049	Bacteria	+	+	+	±	+	+	+	+	+	+	+	+
MCHSJ2	77050	Bacteria	+	+	+	+	+	+	+	+	+	+	+	+
OBR	77052	Bacteria	+	+	+	+	+	+	+	+	+	+	+	+
OBR	77053	Bacteria	+	+	+	+	+	+	+	+	+	+	+	+
OBR	77054	Bacteria	+	+	+	+	+	+	+	+	+	+	+	+
OBR	77055	Bacteria	-	-	-	-	+	+	+	+	+	+	+	+
OBR	77056	Bacteria	+	+	+	+	+	+	+	+	+	+	+	+
OBR	77057	Bacteria	+	+	+	+	+	+	+	+	+	+	+	+
MVUBR	77058	Bacteria	+	+	+	+	+	+	+	+	+	+	+	+
MVUBR	77059	Bacteria	+	+	-	-	+	+	+	+	+	+	+	-
MVUBR	77060	Bacteria	+	+	+	+	+	+	+	+	+	+	+	+
MARM	77061	Bacteria	+	+	+	+	+	+	+	+	+	+	+	+
MARM	77062	Bacteria	+	+	+	+	+	+	+	+	+	+	+	+
MARM	77063	Bacteria	±	-	-	-	+	+	+	+	+	+	+	+
MARM	77064	Bacteria	+	+	+	+	+	+	+	+	+	+	+	+
MARM	77065	Bacteria	+	+	+	-	+	+	+	+	+	+	+	+
MARM	77066	Bacteria	±	±	±	±	+	±	±	±	+	+	+	-
MRAR	77067	Bacteria	+	+	+	-	+	+	+	+	+	+	+	+
MRAR	77068	Bacteria	+	+	+	-	+	+	+	+	+	+	+	+
MVUBR	77071	Bacteria	±	±	-	-	+	+	+	+	+	+	+	+
MVUBR	77072	Bacteria	+	+	+	+	+	+	+	+	+	+	+	+
MVUBR	77074	Bacteria	-	-	-	-	+	+	+	+	+	±	-	-
MCHSJ2	INV PRT215*	Bacteria	+	+	+	+	+	+	+	+	+	+	+	+
MCHSJ2	77076	Bacteria	+	+	+	+	+	+	+	+	+	+	+	+

Sampling site	Consecutive number MHNMC	Type of microorganisms	Tolerance screening on Luria Bertani agar (LB)											
			Cadmium (Cd) mg.L^{-1}				Lead (Pb) mg.L^{-1}				Zinc (Zn) mg.L^{-1}			
			50	150	250	350	50	150	250	350	50	150	250	350
OBR	77077	Bacteria	+	+	+	+	+	+	+	+	+	+	+	+
OBR	77078	Bacteria	+	+	+	+	+	+	+	+	+	+	+	+
OBR	77079	Bacteria	+	+	-	-	+	+	+	+	+	+	+	+
MCHSJ2	77080	Bacteria	±	±	±	±	+	+	+	+	+	+	+	+
MCHSJ2	77081	Bacteria	±	±	±	±	+	+	+	+	+	+	+	+
MCHSJ2	77082	Bacteria	+	+	+	+	+	+	+	+	+	+	+	+
MRAR	77083	Bacteria	+	+	+	+	+	+	+	+	+	+	+	+
MARM	77084	Bacteria	+	-	-	-	+	+	+	+	+	+	+	+
MARM	77085	Bacteria	-	-	-	-	-	-	-	-	-	-	-	-
MARM	77086	Bacteria	+	+	+	-	+	+	+	+	+	+	+	-
MVUBR	77087	Bacteria	±	±	±	±	+	+	+	+	+	+	+	+
MVUBR	77088	Bacteria	+	+	+	-	+	+	+	+	+	+	+	+
MARM	77089	Bacteria	+	+	+	+	+	+	+	+	+	+	+	+
MARM	77090	Bacteria	+	+	+	+	+	+	+	+	+	+	+	+
MARM	77091	Bacteria	+	+	+	+	+	+	+	+	+	+	+	+
MARM	77092	Bacteria	+	+	+	-	+	+	+	+	+	+	+	+
MARM	77093	Bacteria	+	+	+	+	-	-	-	-	+	+	+	+
BSJM2	77094	Bacteria	+	+	+	+	+	+	+	+	+	+	+	+
MVUBR	77096	Bacteria	+	+	-	-	+	+	+	+	+	+	+	+
MVUBR	77097	Bacteria	+	+	+	+	+	+	+	+	+	+	+	+
MVUBR	77098	Bacteria	+	+	-	-	+	+	+	+	+	+	+	+
MCHSJ2	77099	Bacteria	+	+	+	+	+	+	+	+	+	+	+	+
MCHSJ2	77100	Bacteria	+	+	+	+	+	+	+	+	+	+	+	+
MCHSJ2	77101	Bacteria	+	+	+	+	+	+	+	+	+	+	+	+
BSJM2	77103	Bacteria	+	+	+	+	+	+	+	+	+	+	+	+
BSJM2	77104	Bacteria	+	+	+	+	+	+	+	+	+	+	+	+
MPAR	77105	Bacteria	±	-	-	-	+	+	+	+	+	+	+	+
MPAR	77106	Bacteria	+	+	+	+	+	+	+	+	+	+	+	+
MPAR	77107	Bacteria	+	+	+	+	+	+	+	+	+	+	+	+

[*] Catalog number of the Marine Museum of Natural History of Colombia–Makuriwa located in INVEMAR

Supplementary material Table S3. Cadmium (Cd^{2+}), Lead (Pb^{2+}), and Zinc (Zn^{2+}) tolerance screening trials at increased concentrations of Pb2+, Cd2+ and Zn2+ ions of eleven microorganisms. A microorganism was catalogued as tolerant (+) if growth was observed after 24-48 h of culture; not tolerant (-) if growth was inhibited entirely around wells, or sensitive (±) if some colonies were formed (36).

Sampling site	Consecutive numbers MHNMC	Type of microorganisms	Tolerance screening on Luria Bertani agar (LB)									
			Cadmium (Cd) mg.L^{-1}		Lead (Pb) mg.L^{-1}				Zinc (Zn) mg.L^{-1}			
			550	750	450	550	650	750	450	550	650	750
Atrato river mouth	76869	Bacteria	-	-	+	+	+	+	+	+	+	+
	77030	Bacteria	+	+	+	+	+	+	+	+	+	+
	**INV PRT213***	Bacteria	+	+	+	+	+	+	+	+	+	+
San Juan river mouth	76956	Bacteria	-	-	+	+	+	-	+	+	-	-
	77001	Bacteria	+	+	+	+	+	+	+	+	+	+
	77050	Bacteria	+	+	+	+	+	+	+	+	+	+
	**INV PRT215***	Bacteria	+	+	+	+	+	+	+	+	+	+
	77103	Bacteria	-	-	+	+	+	+	+	+	+	+
Baudó river mouth	76952	Bacteria	+	-	+	+	+	-	+	+	-	-
	**INV PRT216***	Bacteria	+	+	+	+	+	+	+	+	+	+
	77058	Bacteria	-	-	+	-	-	-	+	+	+	+

* Catalog number of the Marine Museum of Natural History of Colombia–Makuriwa located in INVEMAR

Supplementary material Table S4. Results obtained by comparing the 16S rRNA sequences to those in the Reference RNA Sequences database of the NCBI.

Catalog number MMNHC*	Description Genera	Accession number GenBank	Query cover	Ident	E-value
INV PRT211	*Stenotrophomonas panacihumi*	NR_117406.1	99%	97.88%	0.0
	Xanthomonas hortensias	NR_181958.1	99%	97.53%	0.0
	Xanthomonas campestris	NR_074936.1	99%	97.53%	0.0
	Xanthomonas arboricola pv. juglandis	NR_113167.1	99%	97.53%	0.0

Catalog number MMNHC*	Description Genera	Accession number GenBank	Query cover	Ident	E-value
	Xanthomonas hortorum pv. gardneri	NR_104793.1	99%	97.53%	0,0
INV PRT212	*Enterobacter sichuanensis*	NR_179946.1	100%	99.68%	0,0
	Enterobacter chengduensis	NR_179167.1	100%	99.68%	0,0
	Enterobacter quasiroggenkampii	NR_179166.1	100%	99.68%	0,0
	Enterobacter bugandensis	NR_148649.1	100%	99.68%	0,0
	Enterobacter mori	NR_146667.2	100%	99.68%	0,0
INV PRT213	*Enterobacter soli*	NR_117547.1	100%	99.15%	0,0
	Huaxiibacter chinensis	NR_184601.1	100%	98.58%	0,0
	Enterobacter asburiae	NR_024640.1	99%	98.28%	0.0
	Klebsiella aerogenes	NR_113614.1	100%	98.15%	0.0
	Klebsiella aerogenes	NR_102493.2	100%	98.15%	0.0
INV PRT215	*Pseudomonas protegens*	NR_114749.1	100%	99.56%	0.0
	Pseudomonas sesami	NR_149822.1	100%	99.34%	0.0
	Pseudomonas saponiphila	NR_116905.1	99%	98.75%	0.0
	Pseudomonas meliae	NR_178423.1	100%	98.67%	0.0

Catalog number MMNHC*	Description Genera	Accession number GenBank	Query cover	Ident	E-value
	Pseudomonas tremae	NR_025549.1	100%	98.67%	0.0
INV PRT216	*Stenotrophomonas maltophilia*	NR_041577.1	100%	99.43%	0.0
	Stenotrophomonas maltophilia	NR_113648.1	100%	99.36%	0.0
	Stenotrophomonas maltophilia	NR_119220.1	100%	99.36%	0.0
	Stenotrophomonas maltophilia	NR_112030.1	100%	99.36%	0.0
	Stenotrophomonas pavanii	NR_116793.1	100%	99.14%	0.0

* Catalog number of the Marine Museum of Natural History of Colombia–Makuriwa located in INVEMAR

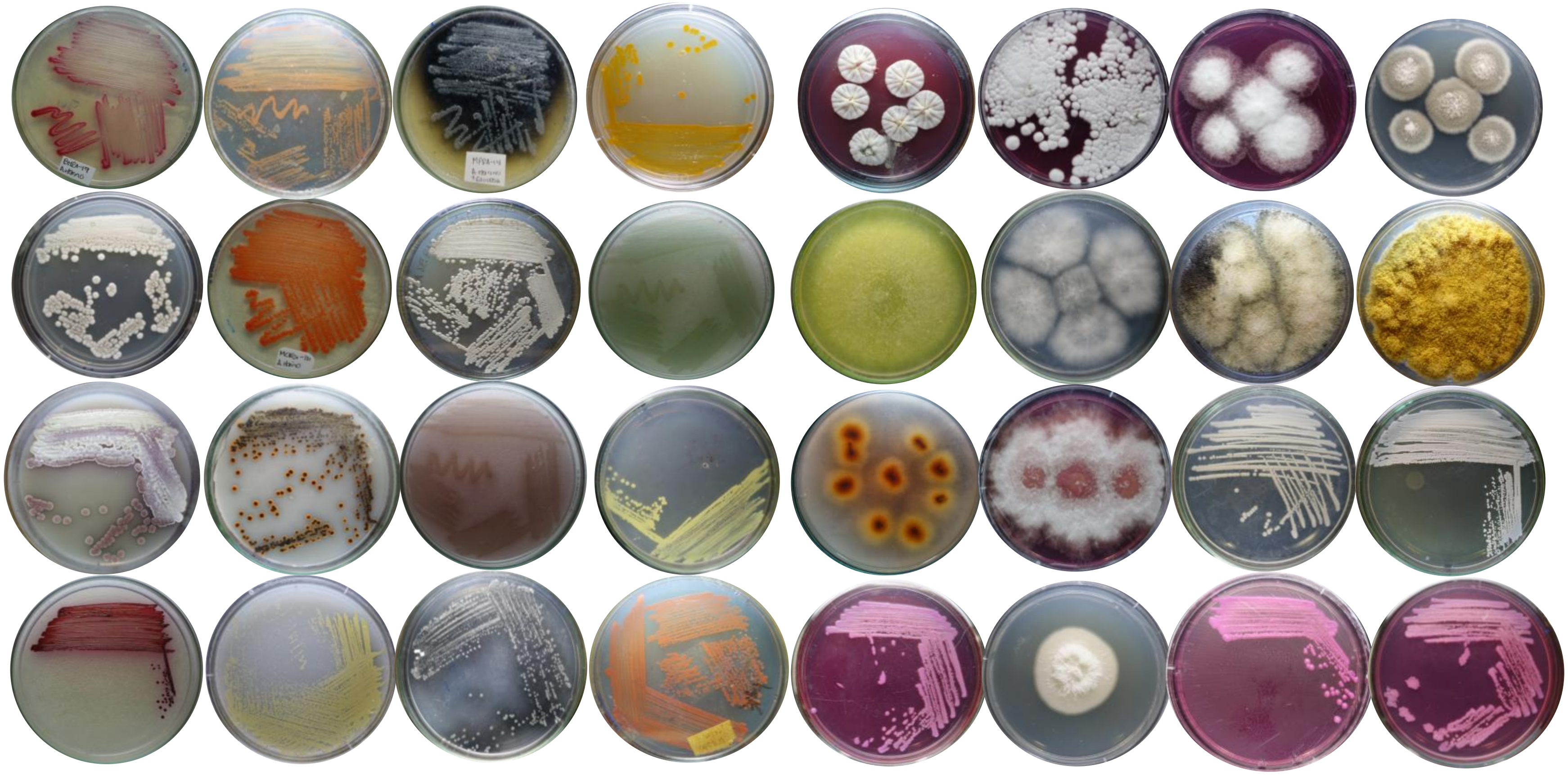

Supplementary material Figure S1. Phenotypic characteristics of some isolated colonies.

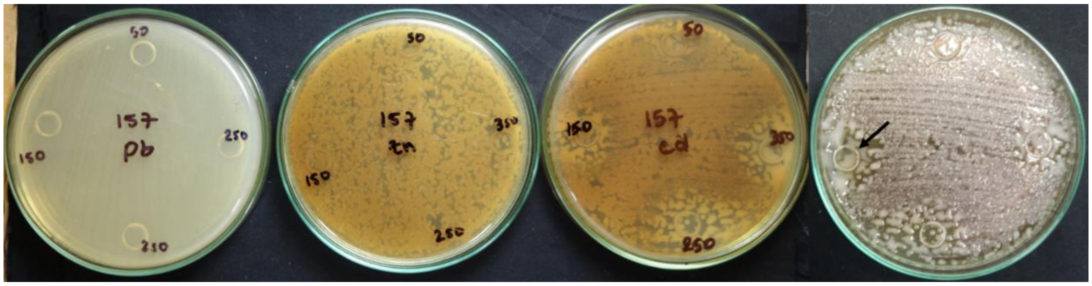

Supplementary material Figure S2. Photographic record of pigments and substances excreted by bacterium 76955 under Cd^{2+} and Zn^{2+} stress conditions.

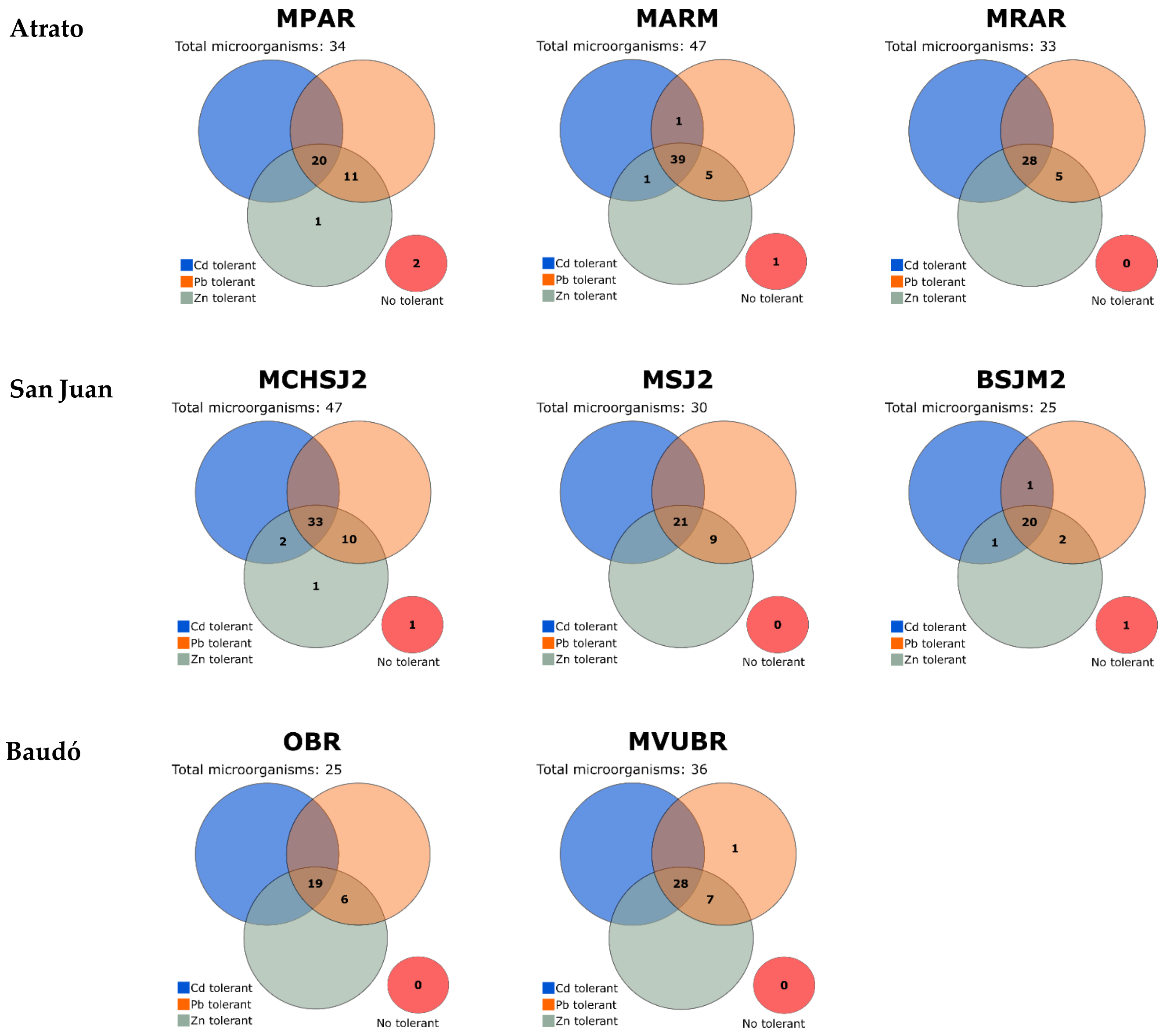

Supplementary material Figure S3. Number of strains tolerant to Cd^{2+}, Pb^{2+}, and Zn^{2+} ions by sampling site. Sampling sites: Atrato river mouth (MPAR, MARM, MRAR), Baudó river mouth (OBR, MVUBR), and San Juan river mouth (MCHSJ2, MSJ2, BSJM2)
